# Immune system modulation & virus transmission during parasitism identified by multi-species transcriptomics of a declining insect biocontrol system

**DOI:** 10.1186/s12864-024-10215-3

**Published:** 2024-03-26

**Authors:** Sarah N. Inwood, Thomas W. R. Harrop, Morgan W. Shields, Stephen L. Goldson, Peter K. Dearden

**Affiliations:** 1 Bioprotection Aotearoa, Genomics Aotearoa, and the Biochemistry Department, University of Otago, Dunedin, New Zealand; 2 Melbourne Bioinformatics, The University of Melbourne, Parkville, VIC 3010 Australia; 3 BioProtection Research Centre, Lincoln University, Lincoln, New Zealand; 4 Biocontrol and Biosecurity Group, AgResearch Limited, Lincoln, Aotearoa, New Zealand

**Keywords:** Multi-species transcriptomics, Endoparasitoid wasp, Parasitism, Host-parasite interaction, Virus transmission, Biocontrol, Resistance

## Abstract

**Background:**

The Argentine stem weevil (ASW, *Listronotus bonariensis*) is a significant pasture pest in Aotearoa New Zealand, primarily controlled by the parasitoid biocontrol agent *Microctonus hyperodae*. Despite providing effective control of ASW soon after release, *M. hyperodae* parasitism rates have since declined significantly, with ASW hypothesised to have evolved resistance to its biocontrol agent. While the parasitism arsenal of *M. hyperodae* has previously been investigated, revealing many venom components and an exogenous novel DNA virus *Microctonus hyperodae* filamentous virus (MhFV), the effects of said arsenal on gene expression in ASW during parasitism have not been examined. In this study, we performed a multi-species transcriptomic analysis to investigate the biology of ASW parasitism by *M. hyperodae*, as well as the decline in efficacy of this biocontrol system.

**Results:**

The transcriptomic response of ASW to parasitism by *M. hyperodae* involves modulation of the weevil’s innate immune system, flight muscle components, and lipid and glucose metabolism. The multispecies approach also revealed continued expression of venom components in parasitised ASW, as well as the transmission of MhFV to weevils during parasitism and some interrupted parasitism attempts. Transcriptomics did not detect a clear indication of parasitoid avoidance or other mechanisms to explain biocontrol decline.

**Conclusions:**

This study has expanded our understanding of interactions between *M. hyperodae* and ASW in a biocontrol system of critical importance to Aotearoa-New Zealand’s agricultural economy. Transmission of MhFV to ASW during successful and interrupted parasitism attempts may link to a premature mortality phenomenon in ASW, hypothesised to be a result of a toxin-antitoxin system. Further research into MhFV and its potential role in ASW premature mortality is required to explore whether manipulation of this viral infection has the potential to increase biocontrol efficacy in future.

**Supplementary Information:**

The online version contains supplementary material available at 10.1186/s12864-024-10215-3.

## Background

The Argentine stem weevil (ASW) *Listronotus bonariensis* (Kuschel) (Coleoptera: Curculionidae) is a significant invasive pasture pest in Aotearoa New Zealand, estimated to cause NZD$200 million in damage p.a. [1], primarily through larval mining in the ryegrass tillers. Neither conventional insecticidal use nor endophytes to deter plant feeding have on their own, provided adequate control over ASW [2–5]. This prompted the extensive use of importation biological control.

The parthenogenetic endoparasitoid wasp *Microctonus hyperodae* (Loan) (Hymenoptera: Braconidae), was collected from the home range of ASW in South America [6, 7], and released throughout New Zealand in the 1990s as a biocontrol agent against ASW [8, 9]. *M. hyperodae* are solitary endoparasitoids, parasitising adult weevils by ovipositing a single egg in the host weevil’s body cavity. Being a koinobiont parasitoid, the host weevil survives *M. hyperodae* larval instar development, before its prepupal emergence which kills the weevil. This life history requires long-lasting manipulation of the weevil’s physiology during parasitism, with parasitoid venom and viruses/virus-like particles particularly important for this in other parasitoid species [10].

Soon after parasitism, ASW are reproductively sterilised by *M. hyperodae*, with some internal organs consumed by the developing larvae (leaving the digestive system and some thoracic tissue intact), and there is a marked reduction in flight capacity [6, 11–13]. Previous research has identified venom components in *M. hyperodae*, with hypothesised links between some of these components and the changes observed in parasitised ASW [14, 15]. Recent work has also demonstrated that *M. hyperodae* carries a novel exogenous dsDNA virus, *Microctonus hyperodae* filamentous virus (MhFV), which is related to another parasitoid-infecting virus that lowers the rate of egg encapsulation during parasitism [16]. Neither the potential role of MhFV in the parasitism of ASW nor how the physiological manipulations of parasitised ASW persist in the six weeks that the parasitoid egg develops within the weevil are currently understood.

Shortly after release, *M. hyperodae* exerted parasitism rates as high as 90% [17–20] which suppressed weevil populations and the associated damage caused by them [17, 21]. Despite such successful establishment and impact of this biocontrol agent, after 14 generations of ASW, *M. hyperodae* parasitism rates had declined significantly. Magnitudes of parasitism rate declines vary, for example by c.60% at Ruakura in the North Island and c.36% at Lincoln in the South Island [13, 22, 23]. No abiotic factors have been found to explain this biocontrol decline, with the only factors that significantly correlate being the number of years after release of the parasitoid, and the number of parasitoid generations since release [23]. Given the intensive selection pressure that high parasitism rates imposed on ASW after *M. hyperodae* release, the high genetic diversity of ASW [24], and the reproductive asymmetry between the sexually reproducing ASW and parthenogenetic *M. hyperodae*, it is thought that ASW may have evolved a heritable parasitism resistance or avoidance strategy [23, 25–27].

Though the evolution of resistance to previously successful importation biological control has not been reported in the field before [28], there are demonstrated examples of host insects evolving a heritable parasitism resistance or avoidance strategy outside of the applied biocontrol context. These strategies can act either before or after the parasitism event occurs. Resistance acting after parasitism often involves an immunological response to the parasitoid egg resulting in its encapsulation and melanisation. This mechanism has been well-characterised in many species such as *Drosophila melanogaster* (reviewed in [29]). Heritable endosymbionts have also been shown to assist in such an immunological response as characterised in the pea aphid *Acyrthosiphon pisum* [30]. However, parasitism resistance can also act to prevent the parasitism event from occurring by increasing host avoidance of the parasite. The field cricket *Teleogryllus oceanicus* is one of the only documented examples of this, having twice independently evolved wing mutations that prevent song production, which its parasitoid fly relies on for host detection [31].

There is no evidence to suggest the decline of ASW parasitism rates is due to immune responses by ASW after parasitism, as contemporary dissections have not detected any encapsulation of *M. hyperodae* eggs [23]. However, laboratory behavioural studies of ASW collected from different regions in New Zealand have identified parasitoid avoidance behaviours exhibited by ASW, which are specific to *M. hyperodae* and influenced by the host plant *Lolium* spp [32–34]. These behaviours are more pronounced in weevils collected from warmer regions where the parasitoid had exerted a stronger selective pressure on ASW, and parasitism rate decline is larger [33]. Avoidance behaviours were not observed in weevils from the Southern region of New Zealand, where parasitism rates have remained low and have not changed significantly (c.8%) [33].

Despite the importance of this biocontrol system to New Zealand’s agricultural economy, relatively little is known about the interactions between ASW and *M. hyperodae* on a genomic or transcriptomic level. With the decline of parasitism rates and thus biocontrol efficacy, improving our understanding of this system is crucial. Here we examine the transcriptional responses of ASW to both parasitism by, and exposure to *M. hyperodae*, to further understand the interactions between these species, and gain insight into the parasitism decline. By performing a multi-species transcriptomic analysis, we also reveal continued expression of *M. hyperodae* venom components inside parasitised ASW, as well as transmission of the recently discovered MhFV [35] to all parasitised ASW and a small number of ASW in which parasitism had not been detected.

## Methods

### Sample collection for ASW microcosm experiment to investigate behavioural avoidance using RNA-seq

ASW were collected from Ruakura and Invermay, New Zealand, these being the same locations that represented the Northern and Southern populations in previous ASW behavioural avoidance studies [33]. The collected weevils were purged of parasitoids for 6 weeks before use in the experiment. As described by Shields et al. (2022) [33] jar microcosms were set up with 10 ASW from the same location and one *M. hyperodae* from Lincoln, New Zealand, on a single *Lolium perenne* ryegrass plant. These microcosms were observed for two hours in a temperature-controlled room, to collect ASW with observed oviposition attempts by *M. hyperodae* (which were interrupted where possible, with these samples considered ‘susceptible’ to parasitism), and ASW exhibiting avoidance behaviour for which oviposition attempts by *M. hyperodae* were not observed (with these samples considered possibly ‘resistant’ to parasitism). This process was repeated until there were 24 weevil samples in each group for each location. Whole ASW were snap-frozen in liquid nitrogen and stored at -80 ℃.

### Sample collection to investigate ASW responses to *M. hyperodae* exposure using RNA-seq

As above, ASW populations were sampled from Ruakura and Invermay, New Zealand, and purged of parasitoids for 6 weeks before use. The parasitoid exposure was then set up in a 60 mm by 15 mm cell culture dish (Cellstar). One ASW, one *M. hyperodae* and one blade of *L. multiflorum* (cv. Tama) ryegrass were then added to these dishes. ASW were exposed to an adult *M. hyperodae* for 30 min, which was observed continuously to ensure that no oviposition attempts occurred. Negative control samples were also established using the same experimental set-up, without the addition of an adult *M. hyperodae* to the dish. Six ASW replicates were collected for each treatment-location pair, resulting in a total of 24 samples. The whole ASW samples were then snap-frozen in liquid nitrogen and stored at -80 ℃.

### Library preparation for RNA-seq

RNA was extracted from weevil samples using a hybrid of Trizol (Ambion) and RNeasy mini kit (Qiagen) methods. This failed to extract adequate RNA for sequencing and quality checks for 17 microcosm samples, reducing the total sample number for this experiment to 79. RNA purity was assessed using a Nanodrop 2000, and RNA integrity was measured using the Agilent 5200 Fragment Analyzer System, with all samples passing these quality assessments. Samples were then prepared for sequencing using the paired-end (2 × 100 bp) Illumina TruSeq mRNA platform and sequenced on an Illumina HiSeq 2500 by Otago Genomics Facility (https://www.otago.ac.nz/genomics/index.html), with the exposure and microcosm experiments sequenced on separate runs.

A multiplex polymerase chain reaction (PCR) test previously established for testing ASW for parasitism by *M. hyperodae* [36] was used to ensure that no exposed ASW were parasitised and to accurately record the parasitism status of microcosm samples. Where extraction yielded enough DNA for both library preparation and the parasitism PCR, RNA was reverse transcribed using a SuperScript VILO cDNA synthesis kit (Thermofisher). The parasitism PCR was carried out on cDNA, using the forward primer C1-J-1718 as a positive control which amplifies for both ASW and *M. hyperodae*, while COIfwdMax is specific to *M. hyperodae*, both using the reverse primer C1-N-2191. PrimeSTAR HS DNA Polymerase premix (Takara) was used to set up 12.5 uL PCR reactions, with 1.25 uL of cDNA, and 10X primer mix as per the published protocol. Samples were run alongside a positive control with *M. hyperodae* DNA, and results were examined on a 2% agarose (low EEO, AppliChem) gel stained with ethidium bromide.

### Pre-processing of RNA-seq samples for transcriptome assembly

BBDuk v38.00 [37] was used to quality trim and remove Illumina sequencing adapters, using default settings with trimq set to 15. FastQC v11.9 was then used to ensure quality trimming was successful and no further issues remained with samples. Kraken2 v2.1.2 [38] was then used to taxonomically classify reads against the Kraken standard database (downloaded 17th May 2021), and to identify potentially contaminated samples.

### *De novo* ASW transcriptome assembly and quality assessment

Transcriptome assembly used only the reads from the ASW parasitoid exposure experiment, as quality-control results indicated these samples generated adequate coverage. Before assembly, BBMerge v38.00 [39] was used to merge overlapping reads using the ‘very strict’ setting. *De novo* transcriptome assembly was performed with Trinity v2.11.0 [40] using default settings with output from BBMerge. Transcript redundancy was reduced by retaining only the longest transcript assembled for each gene. BUSCO v5.2.1 [41] was then used to assess transcriptome completeness using the endopterygota_odb10 lineage. The scripts used for this pipeline are available at https://github.com/sarahinwood/asw-transcriptome. The transcriptome assembly was annotated using the Trinotate pipeline v3.2.0 as was used for the previous *M. hyperodae* transcriptome assembly [15], generating BlastX, BlastP, Pfam, Kegg and GO annotations.

### RNA-seq triple species read mapping & power analysis

All RNA-seq samples were quasi-mapped using Salmon v1.5.1 [42] with default settings, against a concatenated file containing the length-filtered ASW transcriptome, as well as the length-filtered *M. hyperodae* transcriptome (with any significant hits in the *M. hyperodae* transcriptome to MhFV genes removed) and predicted genes from the MhFV genome from previous work [15, 35]. DESeq2 v1.34 [43] was used to create the DESeqDataSet (DDS) object, by importing Salmon output files using tximport v1.22 [44] in R v4.1.3 [45]. Size factors were estimated on the whole concatenated file, before being split into three separate DDS objects containing ASW, *M. hyperodae* and MhFV genes for analysis of each species separately.

A blind variance stabilising transformation (VST) was performed on the DDS objects, which were used for principal components analysis (PCA). PCA on ASW gene expression revealed a large split on principal component 1 (PC1), with investigation of the 500 genes contributing to PC1 and a pairwise comparison between samples on either extreme revealing this to be ASW sex. ASW sex was controlled for in all future DESeq2 and DEXSeq analyses, using the sign of PC1.

A power analysis was carried out to determine the ability of differential expression to be detected in comparisons, with multiple log_2_ fold-change (LFC) thresholds. Power estimations were calculated using RnaSeqSampleSize v2.2 [46] with LFC thresholds of 2 and 5, and a repNumber of 10,000. As no currently available power estimation tool can use both user-provided count data and perform analysis on factors with more than two levels, power estimations for analyses involving such factors or an interaction term (which results in at least four sample groups) were performed by iteratively comparing one sample group of interest to all others.

### Differential gene expression analysis

Significant differentially expressed genes (DEGs) were identified using Wald tests in DESeq2 v1.34 with designs as specified below, a log_2_ fold-change magnitude threshold of 1, and an alpha threshold of 0.05. The effects of parasitism were investigated, on the ASW transcriptome, *M. hyperodae* transcriptome with MhFV genes removed, and MhFV genes, using the design ∼ ASW_location + PC1 + parasitism_status. Location-specific responses were not investigated due to the low number of Ruakura parasitised samples and lack of time-point standardisation.

Further analyses were then performed on the ASW transcriptome to investigate potential resistance mechanisms explaining biocontrol decline. Comparison of ASW from the Southern Invermay population, where parasitism rates have not declined significantly, and the Northern Ruakura population, where the magnitude of parasitism rate decline is large, used the design ∼ parasitism_status + PC1 + ASW_location. The pairwise comparison between the exposure/control treatments used the design ∼ PC1 + ASW_location + exposure. Investigation of location-specific responses to exposure used the design ∼ PC1 + ASW_location + exposure + ASW_location: exposure. The pairwise comparison between ASW that had an oviposition attempt by *M. hyperodae* (both observed and interrupted or successful parasitism) and those where no attempt was observed used the design ∼ PC1 + parasitism_status + ASW_location + oviposition_attempt_status. To investigate an interaction between location & oviposition attempt status the design ∼ PC1 + parasitism_status + ASW_location + oviposition_attempt_status + ASW_location: oviposition_attempt_status was used.

Significant DEG lists were briefly investigated in full, before being filtered to keep only DEGs with a mean transcripts per million (TPM) value above five in at least one experimental group, to remove DEGs where significance was driven by a single sample with low expression. For all results, any DEGs without Trinotate BlastX annotation (from the UniProtKB/SwissProt database) were subject to another BlastX v2.13 search against the non-redundant database (downloaded on 20th March 2023). Results were first filtered to remove uncharacterised or hypothetical annotations before the hit with the lowest E-value (and highest bit-score in the case of a tie) was retained. Heatmaps of expression were generated using VST normalized data with pheatmap v1.0.12 [47]. The scripts used for this differential expression analysis are available at https://github.com/sarahinwood/asw-rnaseq-paper.

### Differential exon usage analysis

As well as investigating changes in expression on the gene level, we investigated changes on the transcript level via differential exon usage (DEU) analysis, as changes in exon/transcript usage don’t always result in differential expression on the gene level. Supertranscripts, containing all unique exons from each assembled Trinity ‘gene’, were assembled for both the ASW and *M. hyperodae* transcriptomes using the Trinity v2.11.0 SuperTranscripts DEXSeq wrapper script, and then a file containing supertranscripts from both species, as well as predicted genes from MhFV was created. This was used for DEU analysis, which involved mapping reads to the supertranscripts using STAR v2.7.2b [48] as per the Trinity SuperTranscripts DEXSeq wrapper script, before analysis with DEXSeq v1.40.0 [49].

For DEXSeq analysis, size factors were first estimated on all supertranscripts plus MhFV genes, before being split into species-specific files. Further DEXSeq analysis was only carried out using the ASW supertranscripts, first filtered to keep only exons with at least ten counts in half of the smallest experimental group in the comparison/dataset (25 for the full location dataset, three for the exposure dataset, and six for the microcosm dataset). This filtering removed exons not expressed in most samples, thus reducing computational time while also generating more accurate results [50].

Samples from both datasets were used to investigate DEU between locations (with the design ∼ sample + exon + PC1:exon + Location:exon). The microcosm dataset was investigated using a pairwise comparison for parasitism status (∼ sample + exon + PC1:exon + parasitism:exon) and oviposition attempt status (∼ sample + exon + PC1:exon + oviposition_attempt_status:exon). Analyses to investigate location-specific effects of oviposition attempt status was performed as pairwise comparisons for each location separately using the same design, as DEXSeq requires the interaction term for analysis to be the factor of interest:exon, preventing a location-oviposition attempt interaction analysis.

Pairwise tests were carried out on the exposure experiment samples to identify DEU resulting from exposure treatment (with the design ∼ sample + exon + PC1:exon + exposure:exon). Analysis to identify location-specific responses to exposure were performed as pairwise comparisons for exposure for each location separately. Reduced models for all analyses, required for DEU testing, were the same models used for design minus the final interaction term of interest. The scripts used for this DEU analysis are available at https://github.com/sarahinwood/asw-deu.

### Gene ontology overrepresentation analysis

ClusterProfiler v4.2.2 [51] was used to investigate the overrepresentation of Pfam domains or BlastP-derived gene ontology (GO) terms in significant DEG or DEU lists compared to the whole transcriptome. This was performed on full DEG lists before TPM filtering, and for both DEG and DEU lists was only performed where there were more than three genes with Pfam or GO annotation.

### Variant calling and GWAS analysis

Variants were called using all RNA-seq samples against the combined SuperTranscripts file with the Trinity wrapper script ‘SuperTranscripts/AllelicVariants/run_variant_calling.py’ which uses GATK Haplotype Caller v4.1.4.0 [52]. This generated vcf files for each sample, which were then merged with bcftools v.1.9.1, keeping only variants that passed the filtering steps in the Trinity wrapper. As per GATK best practices [53, 54] GATK Variant Filtration v4.1.4 was then used to filter variants, to keep those with a depth of coverage above 10 and a StrandOddsRatio less than 3. The vcf file was then filtered to retain only ASW single nucleotide polymorphisms (SNPs), removing those with less or more than 2 alleles, those within 10 bp of an insertion or deletion, and all singletons.

The filtered SNP set was used for PCA with Plink v2.0 [55], both before and after the SNP set was pruned for sites in linkage disequilibrium. GWAS analyses were performed using Plink v2.0 with the glm command, with PCA eigenvectors used as covariates, to detect any SNPs significantly associated with ASW oviposition attempt and parasitism statuses (with adjusted p-value threshold of 0.05). Analyses were performed on the unpruned and pruned SNP sets, as well as on the Ruakura and Invermay ASW SNP sets separately. Scripts for variant calling are available at https://github.com/sarahinwood/asw-variants-rnaseq and scripts for the GWAS analysis are available at https://github.com/sarahinwood/asw-plink-gwas.

## Results

### ASW transcriptome assembly

To assess the impact of parasitism and parasitoid exposure on ASW gene expression we first assembled a reference transcriptome for ASW. Sequencing from the exposure samples generated 25.9–40.9 million reads per sample, while the microcosm samples had 10.8–16.9 million reads per sample, with trimming retaining over 99.9% of reads. *De novo* assembly with Trinity used 812.9 M reads from the exposure experiment samples, with 70.5% of these reads merged prior to assembly. Trinity assembled 254,561 transcripts sorted into 126,162 ‘genes’ with a GC content of 33.6%. BUSCO analysis indicates that our transcriptome has a high level of completeness though many BUSCO genes were duplicated (C:98.4% [S:27.3%, D 71.1%] F: 0.4%, M:1.2%). This was reduced when the assembly was filtered to retain only the longest isoform per gene (C:96.5% [S:95.7%, D:0.8%), F:0.8%, M:2.7%) indicating BUSCO redundancies were predominantly due to assembly of multiple transcript isoforms per gene. Therefore, further analyses used a fasta file with the longest isoform per gene only. A BlastX search against the UniProtKB/Swiss-prot database as part of the Trinotate pipeline found significant hits for 11.7% of Trinity genes. Transdecoder predicted complete coding sequence in 13.8% of genes which had an average length of 2049 bp. With only a further 2841 genes without Transdecoder predictions over 2000 bp, the remaining genes likely lack predictions as they were incompletely assembled. Transdecoder predictions were used for a BlastP search that found significant hits for 9.3% of Trinity genes (71.8% of those with Transdecoder predictions). Significant hits to Pfam protein domains were found for 9.8% of genes, and GO terms associated with BlastP hits were found for 9.1% of genes.

### Parasitism status & triple species RNA-seq mapping

To confidently identify changes in ASW gene expression associated with parasitism and factors of interest, it was first necessary to confirm the parasitism status of each sample. A parasitism PCR test detected no parasitism in samples from the exposure experiment and detected parasitism in 19 of the 59 microcosm samples that amplified successfully. Given these results, and the potential for transmission of MhFV by *M. hyperodae* during parasitism, RNA-seq read mapping was performed against a file containing the length-filtered *M. hyperodae* and ASW transcriptomes, as well as predicted gene sequences from MhFV. To confirm the parasitism status of samples, DESeq2 normalised counts against the parasitism PCR target transcript (*M. hyperodae* TRINITY_DN7604_c0_g1) were used, confirming all successful PCR results, and revealing a further two parasitised samples for which the PCR test had failed (Supplementary Fig. 1).

This detection of parasitism led to a re-classification of several samples from their original groups. Parasitism was detected in 40% of Invermay ASW originally classified as ‘resistant’ due to exhibiting avoidance behaviours with no observed oviposition attempts by *M. hyperodae*. Parasitism was also detected in ‘susceptible’ ASW (where oviposition attempts were observed and interrupted to prevent successful parasitism), for 42.9% of Invermay and 20% of Ruakura ‘susceptible’ ASW. Importantly, no parasitism was detected in Ruakura ‘resistant’ ASW that exhibited evasive behaviours. Sample groups were therefore changed to ASW that had oviposition attempts (with two subgroups, either interrupted and not parasitised, or successfully parasitised), and ASW that exhibited evasive behaviours, had no oviposition attempt observed, and in which parasitism was not detected (Supplementary Table 1).

Overall sample mapping rates varied between 84.8% and 91.9% with a mean of 89.5%. Comparing the percentage of mapped reads that mapped to each species between parasitised and unparasitised samples revealed a significant difference for all species (ASW parasitised vs. unparasitised: 88.3% (10.8 million reads) vs. 96% (12.1 million reads), *p*-value = 1.6E-03; *M. hyperodae*: 11.6% vs. 3.9%, *p*-value = 1.8E-03; MhFV: 0.14% vs. 0.01%, *p*-value = 7.9E-08). Significantly higher read mapping to MhFV in parasitised ASW indicates it is likely that MhFV is transmitted to ASW during parasitism. Any reads mapping to *M. hyperodae* in samples where parasitism could not be detected were considered mis-mapped.

These mapping results are consistent with those from gene expression PCA for *M. hyperodae* and MhFV, which both showed strong sample grouping based on parasitism status (Supplementary Fig. 2). ASW PCA revealed strong clustering in PC1 which was not explained by any experimental variables, with PC2 explained by parasitism status (Supplementary Fig. 2A). Investigation of annotations for the 500 genes used for PCA revealed this clustering was likely a result of ASW sex (determined by investigation of gene functions, as samples were not sexed before extraction), which was therefore controlled for in all further analyses (using the sign of ASW PC1).

In RNA-seq experiments low sample numbers and low sequencing depth both reduce the power to detect differential expression with low sequencing depth reducing the likelihood of sequencing genes with lower expression levels [56]. There is a trade-off between sample replicate numbers and sequencing depth, with previous work showing that increasing replicate numbers generally has a larger impact on increasing power [57, 58]. We therefore prioritised high sample numbers over increased read depth, particularly in the microcosm experiment. We then performed power analyses to ensure that where little to no differential gene expression was detected, this was due to a lack of difference in gene expression levels rather than an underpowered experiment. For the ASW exposure experiment results indicated there was power to detect 37.84–40.20% of DEGs with a log_2_ fold-change threshold of two, while the power to detect larger fold changes was much higher, with 89.99–91.14% of DEGs detected with log_2_ fold-change thresholds of five (Supplementary Table 2). The microcosm and location analyses have far higher power because of increased sample sizes, with power to detect 89.14–92.52% of DEGs with a log_2_ fold-change threshold of two, and 99.66–99.98% with a log_2_ fold-change threshold of five. These experiments have enough power to detect large changes in gene expression, with the microcosm and location analyses also well-powered to detect smaller changes in expression. Therefore, in differential expression analyses that return a low number or no DEGs, there is a reasonable level of confidence that this is due to a lack of differential expression.

### Transcriptomic analyses of ASW after parasitism reveal metabolism and immune system manipulation

**Fig. 1:**
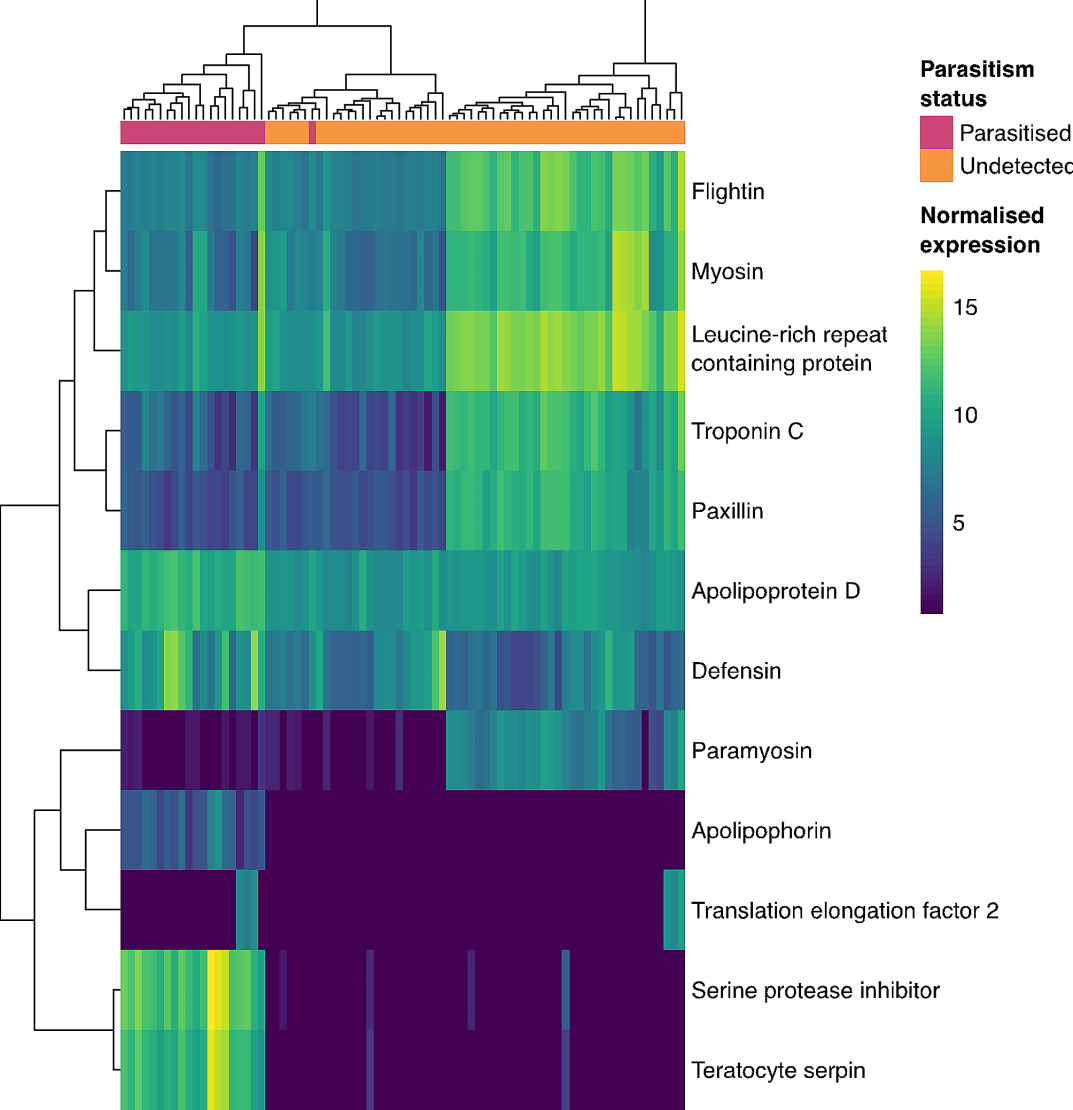
ASW genes significantly differentially expressed after parasitism. A clustered heatmap showing VST normalized expression of DEGs with a mean TPM value above five and BlastX annotation. The parasitism status of each ASW sample is indicated in pink and orange boxes

To identify the effects of parasitism on ASW gene expression, a pairwise comparison was performed between parasitised samples and those in which parasitism was not detected. This analysis found 21 DEGs, 18 of which were retained after TPM filtering, which removed genes that had a low level of expression and/or were only expressed in a small subset of samples and were therefore not of interest. Of these 18 DEGs, 12 had significant BlastX annotations, with six of these annotated DEGs upregulated in parasitised ASW, while six were downregulated (Table 1; Fig. 1). DEXSeq analysis detected significant DEU in 336 exons in 200 genes, 118 of which had Trinotate BlastX annotation (Supplementary Table 3), with five of the same genes also significantly differentially expressed in the DESeq2 analysis.

**Table 1:** ASW genes significantly differentially expressed in the parasitism analysis and with BlastX annotation

Trinity Gene ID	LFC	Padj	BlastX Annotation	Sequence identity (%)	BlastX E-value	Parasitised TPM	Unparasitised TPM
TRINITY_DN46396_c0_g1	23.55	8.42E-07	translation elongation factor 2-like (*Agrilus planipennis*)	91.67	9.57E-38	44.12	23.22
TRINITY_DN35637_c0_g1	14.48	5.23E-49	serine protease inhibitor 3/4-like isoform X5 (*Megalopta genalis*)	45.69	7.75E-20	3885.44	0.22
TRINITY_DN35637_c0_g2	14.13	2.20E-62	teratocyte serpin II (*Cotesia flavipes*)	47.22	8.95E-18	8325.17	0.41
TRINITY_DN78148_c0_g1	8.11	1.85E-30	Apolipophorin (*Cotesia flavipes*)	67.78	4.86E-35	49.59	0
TRINITY_DN3215_c1_g1	3.45	3.47E-03	defensin, isoforms B and C (*Sitophilus oryzae*)	72.73	3.97E-33	873.85	204.45
TRINITY_DN3452_c0_g1	1.85	6.99E-03	apolipoprotein D-like isoform X1 (*Anthonomus grandis grandis*)	81.85	5.05E-156	203.50	51.32
TRINITY_DN6567_c0_g1	-3.10	3.41E-02	putative leucine-rich repeat-containing protein DDB_G0290503 (*Anthonomus grandis grandis*)	77.38	0	13.91	96.26
TRINITY_DN3087_c3_g1	-3.24	2.44E-02	flightin isoform X2 (*Anthonomus grandis grandis*)	81.08	8.15E-54	59.13	388.52
TRINITY_DN3943_c0_g1	-3.55	2.68E-02	troponin C, isoallergen Bla g 6.0101-like isoform X1 (*Sitophilus oryzae*)	90.85	1.66E-95	15.17	179.43
TRINITY_DN18461_c0_g1	-3.96	2.09E-04	myosin light chain alkali-like (*Sitophilus oryzae*)	78.85	4.84E-64	188.00	772.60
TRINITY_DN10508_c0_g1	-4.59	1.26E-07	paxillin isoform X1 (*Dendroctonus ponderosae*)	57.88	2.06E-116	2.97	68.54
TRINITY_DN6332_c1_g1	-7.30	6.63E-08	paramyosin, short form-like (*Dendroctonus ponderosae*)	58.59	3.90E-87	0.07	14.85

DESeq2 Log_2_ fold-changes (LFC) and adjusted P-values (Padj) are reported from DESeq2 analysis, BlastX annotation is from a search against the non-redundant database, and mean TPM values for experimental groups were calculated from Salmon output. A positive LFC indicates upregulated expression in ASW that were parasitised by *M. hyperodae*

Sample clustering on the VST normalised heatmap shows two separate clusters of ASW where parasitism was not detected, as well as the parasitised ASW samples (Fig. 1). This is due to expression patterns of some genes which had higher expression in some ASW samples where parasitism was not detected, and lower expression in both parasitised samples and other samples where parasitism was not detected. This patterns is not explained by any experimental variables, with both ASW source location and PC1 (as a proxy for ASW sex) controlled for in this analysis. This may be due variation between samples in another variable for which data was not collected e.g. ASW age. While these genes are significantly differentially expressed, the biological significance of DEGs with these expression patterns is less clear.

#### Innate immune response

One gene both significantly upregulated in parasitised ASW and with significant DEU for four exons is TRINITY_DN3215_c1_g1, annotated as the antimicrobial peptide (AMP) defensin (Table 1, Supplementary Table 3). Defensins are a group of antimicrobial peptides (AMPs) produced in response to injury [59]. Upregulation of AMP production has been observed in multiple host-parasitoid systems after parasitism [60, 61]. This upregulation by parasitised ASW is likely either a response to the wound created by the *M. hyperodae* ovipositor during parasitism, or to immune system manipulation caused by *M. hyperodae* during parasitism.

Another two DEGs, TRINITY_DN35637_c0_g1 and TRINITY_DN35637_c0_g2 which were both upregulated after parasitism, were annotated as serine protease inhibitors (serpins) (Table 1). Serpins regulate the insect humoral innate immune response, initiating responses such as AMP production through the Toll pathway [62–64]. Serpins have also been shown to negatively regulate the prophenoloxidase (PPO) cascade required for a melanisation immune response towards a parasitoid egg [65–67]. Some parasitoid wasps cause downregulation of host serpin expression to manipulate the host cellular immune system [68], and have serpins in their venom [69–71], though here serpins are upregulated in parasitized ASW. This serpin upregulation may therefore link to AMP production, rather than to manipulation of the ASW cellular immune system. An upregulation of serpin gene expression after parasitism, alongside an increase in AMP production, has been observed in other host-parasitoid systems [e.g. 61, 72, 73].

There are also several genes involved in the innate immune system that had significant DEU but were not differentially expressed in DESeq2 analysis (Supplementary Table 3). This includes an exon in a gene annotated as the AMP coleoptericin (TRINITY_DN1751_c0_g2) which had significantly upregulated expression after parasitism, two genes annotated as serine proteases (TRINITY_DN1306_c0_g1 and TRINITY_DN1694_c0_g1) containing four exons which have significantly upregulated expression after parasitism, and one gene with significantly downregulated expression of an exon after parasitism that is annotated as a serine protease inhibitor (TRINITY_DN2262_c0_g1), which negatively regulates the melanisation response in *D. melanogaster* [74].

#### Lipid transport

Two DEGs were involved in lipid transport, both of which were upregulated in parasitized ASW. TRINITY_DN78148_c0_g1 is annotated as Apolipophorin-III (ApoLp-III) (Table 1) which is an important component of plasma hormone-induced lipid mobilization insects, necessary when large amounts of lipids are transported [75]. ApoLp-III is upregulated in parasitized *Plutella xylostella* plasma [68], and induces AMP expression in *Hyphantria cunea* [76], with other research suggesting a link between ApoLp-III and the innate immune system. TRINITY_DN3452_c0_g1, which is both significantly upregulated following parasitism (Table 1) and has significant DEU (Supplementary Table 3), is annotated as Apolipoprotein D, also involved in cholesterol binding and lipid transport [77].

Several genes with annotations related to lipid transport had significant DEU but were not detected in the DESeq2 analysis (Supplementary Table 3). Genes with exons significantly upregulated after parasitism have annotations such as the perilipin Lsd1 (TRINITY_DN5777_c0_g1) which facilitates lipolysis and regulates lipid storage in insects [78, 79], Seipin (TRINITY_DN1857_c0_g1) which regulates lipid storage via a calcium-dependent mechanism [80], and a transfer protein (TRINITY_DN878_c0_g1) that is required for assembly and secretion of plasma lipoproteins [81]. Genes with exons significantly downregulated after parasitism have annotations such as a second Apolipoprotein D (TRINITY_DN2901_c0_g1), and a transfer protein from *Danio rerio* required for assembly and secretion of plasma lipoproteins (TRINITY_DN878_c0_g1) [81].

#### Glucose metabolism & transport

While no genes with differential expression following parasitism were involved in glucose metabolism, several genes with significant DEU were (Supplementary Table 3). A gene annotated as glucose 6-phosphate dehydrogenase (TRINITY_DN1863_c0_g1, G6PD) had an exon upregulated following parasitism. G6PD is a rate-limiting enzyme that catalyses the first step of the pentose phosphate pathway [82]and was the only gene in this pathway with significant DEU. Two genes annotated as Facilitated trehalose transporter Tret1 (TRINITY_DN3951_c0_g1 and TRINITY_DN20790_c0_g1) had two exons significantly upregulated following parasitism. Trehalose is the main hemolymph sugar in most insects, with Tret1 required for the transport of trehalose from the fat body, where it is synthesised, into other tissues [83].

Three genes with annotations involved in glucose metabolism had significant downregulation of an exon (Supplementary Table 3). The first is pyruvate kinase (TRINITY_DN640_c0_g1) which catalyses the last step of glycolysis and is significantly downregulated at the gene expression level in *Sarcophaga bullata* when parasitised by *Nasonia vitripennis* [60]. The second is a gene annotated as UTP-glucose-1-phosphate uridylyltransferase (TRINITY_DN6632_c1_g1) which is involved in trehalose metabolism and in forming a precursor for glycogen biosynthesis [84]. The final gene with significant DEU due to downregulation of an exon after parasitism is Fructose 1,6-bisphosphatase (TRINITY_DN4956_c0_g1), which catalyses the formation of fructose 6-phosphate in gluconeogenesis. Upregulation of Fructose 1,6-bisphosphatase on a gene expression level after parasitism has been observed in *Ostrinia furnacalis* and *Diatraea saccharalis* [85, 86], though the implications of exon downregulation after parasitism are unclear.

#### Muscle components

Five of the seven annotated DEGs downregulated after parasitism are muscle components (flightin, troponin C, myosin, paxillin and paramyosin) (Table 1). The same genes annotated as flightin and troponin C also had significant DEU detected due to exon downregulation after parasitism, as did different Trinity genes sharing the same annotations (Supplementary Table 3). Flightin is an indirect flight muscle-specific protein, required for myosin assembly during development, with null mutants unable to fly and experiencing progressive flight muscle loss as adults [87, 88]. Paramyosin and myosin are both structural muscle proteins, that play a role in flight muscles in *D. melanogaster* among other tissues [89, 90]. Troponin C is a tubular muscle component in *D. melanogaster*, again with roles in flight muscles among other tissues [91, 92]. Paxillin is an adapter protein involved in actin-membrane attachment, which controls the size of some muscles in *D. melanogaster* [93, 94].

DEXSeq analysis also detected DEU in other genes involved in muscle development (Supplementary Table 3). There was decreased expression of six exons and increased expression of one exon in a gene annotated as a Muscle LIM protein (TRINITY_DN7066_c0_g1), which maintains muscle structural integrity and regulates actin cross-linking [95] with null mutants in *D. melanogaster* unable to fly or beat their wings [96]. There is also significant downregulation of two exons and upregulation of one exon in a gene annotated as Cappuccino (TRINITY_DN4199_c1_g1), which nucleates actin filaments and is required for the building of an actin mesh during oogenesis in *D. melanogaster* [97], with mutation of Cappuccino resulting in female sterility [98].

ClusterProfiler analysis on the parasitism DEG list detected significant enrichment of 38 GO terms and nine Pfam domains (Supplementary Fig. 3), though enrichment of all Pfam domains and all but four GO terms were driven by a single DEG, with the other four driven by two to three DEGs. The GO term overrepresentation primarily highlights changes in muscle organisation, while most Pfam enrichment is due to multiple EF-hand domains. ClusterProfiler analysis on the parasitism DEU list detected significant enrichment of two Pfam domains (PF13499.6 and PF13833.6, both EF-hand domains) driven by the same 5 genes with DEU, and three GO terms, two of which relate to glucose metabolism (Supplementary Fig. 4).

No analysis was performed to look for location-specific responses to parasitism due to the lack of standardised time points since parasitism, and the low number of parasitised Ruakura ASW. Biocontrol decline may involve ASW responses after parasitism leading to the survival of parasitised ASW, which would not have been detected without a location-specific analysis and timepoints during parasitism, though no change in encapsulation rates of *M. hyperodae* eggs by ASW has been detected in dissections over time [23].

### Transmission of MhFV to parasitised ASW

**Fig. 2:**
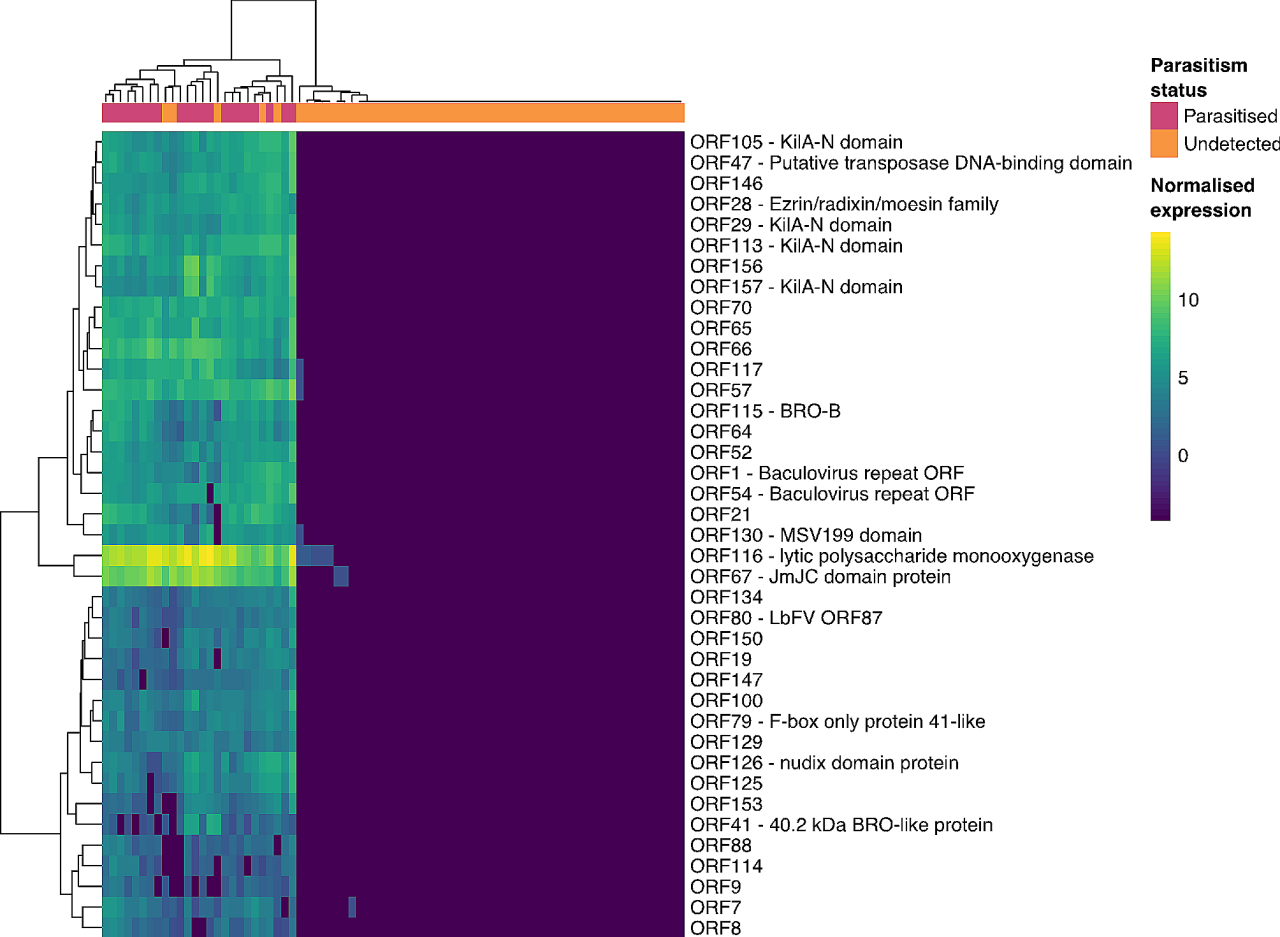
MhFV genes significantly expressed in parasitised ASW. A clustered heatmap showing VST normalized expression of DEGs with a mean TPM value above five. BlastX or Pfam annotation is included where available. The parasitism status of each ASW sample is indicated in pink and orange boxes

A pairwise comparison with this dataset based on parasitism status revealed transmission of MhFV to all parasitised ASW (Fig. 2). Of the total 158 predicted genes in the MhFV genome, 118 are significantly differentially expressed in parasitised ASW (Supplementary Table 4), which could reflect expression within either the oviposited *M. hyperodae* egg or within ASW tissues themselves. Of these DEGs, 40 had a TPM value above five, and 23 of these also had a Blast or Pfam annotation from a previous analysis [35]. ORF116 had the highest TPM at 758.95 and is annotated as a lytic polysaccharide mono-oxygenase, which may act to facilitate the spread of the viral infection [35]. ORF67 had the second highest TPM for an annotated gene at 87.60 and is annotated as a Jmjc-domain containing protein, involved in transcriptional regulation with a potential eukaryotic origin in LbFV-like viruses [35, 99].

Other MhFV genes expressed in parasitised ASW with TPMs above five include several containing Bro-N or KilA-N domains, and one with a nudix domain. These domains have been found in a wide range of eukaryotic viruses, though their functions in viruses have not been well characterised. Both BRO genes and the KilA-N domain have been demonstrated to bind DNA, with BRO genes also able to bind RNA and hypothesised to potentially alter DNA replication or host transcription [100, 101]. Ac38 is a nudix-domain containing gene in *Autographa californica* multiple nucleopolyhedrovirus which is required for virus production [102], and may act as an mRNA decapping enzyme to negatively regulate gene expression [103].

This analysis also highlighted five samples, four from Invermay and one from Ruakura, in which no parasitism was detected either through PCR or sequencing, that also had MhFV gene expression (Fig. 2), with TPMs ranging from 464 to 0.0 with a mean TPM of 7.1. Two of these samples had observed oviposition attempts that were interrupted before oviposition could be completed successfully. This indicates that interrupted/unsuccessful parasitism attempts by *M. hyperodae* may lead to the transmission of MhFV to unparasitised ASW. However, MhFV expression was not observed in the other 26 ASW with manually interrupted parasitism attempts. The remaining three samples did not have observed oviposition attempts, so virus transmission may have been due to an unsuccessful oviposition attempt that was not observed, or an alternate transmission route. MhFV expression was not detected in any ASW from the exposure experiment that was observed closely and never had any parasitism attempts, nor in the ASW that were never exposed to *M. hyperodae*. A pairwise comparison of ASW gene expression between unparasitised ASW with and without a detected MhFV infection revealed six DEGs indicating infection affects ASW gene expression. However, none of these genes had significant BlastX hits or predicted protein domains from which to infer putative functions.

### Continued *M. hyperodae* venom expression in parasitised ASW links to host manipulations and viral infection

A pairwise comparison of parasitised ASW and those in which parasitism was not detected was also performed against the *M. hyperodae* transcriptome. Due to the lack of parasitism time-points, this detects all *M. hyperodae* expression in parasitised ASW rather than just *M. hyperodae* genes upregulated after parasitism. This detected 11,590 DEGs, all but one of which had higher expression in parasitised ASW as was to be expected, with the one gene with lower expression in *M. hyperodae* a result of mis-mapped reads, as unparasitised ASW samples should not have higher expression of any *M. hyperodae* genes than parasitised ASW.

**Fig. 3:**
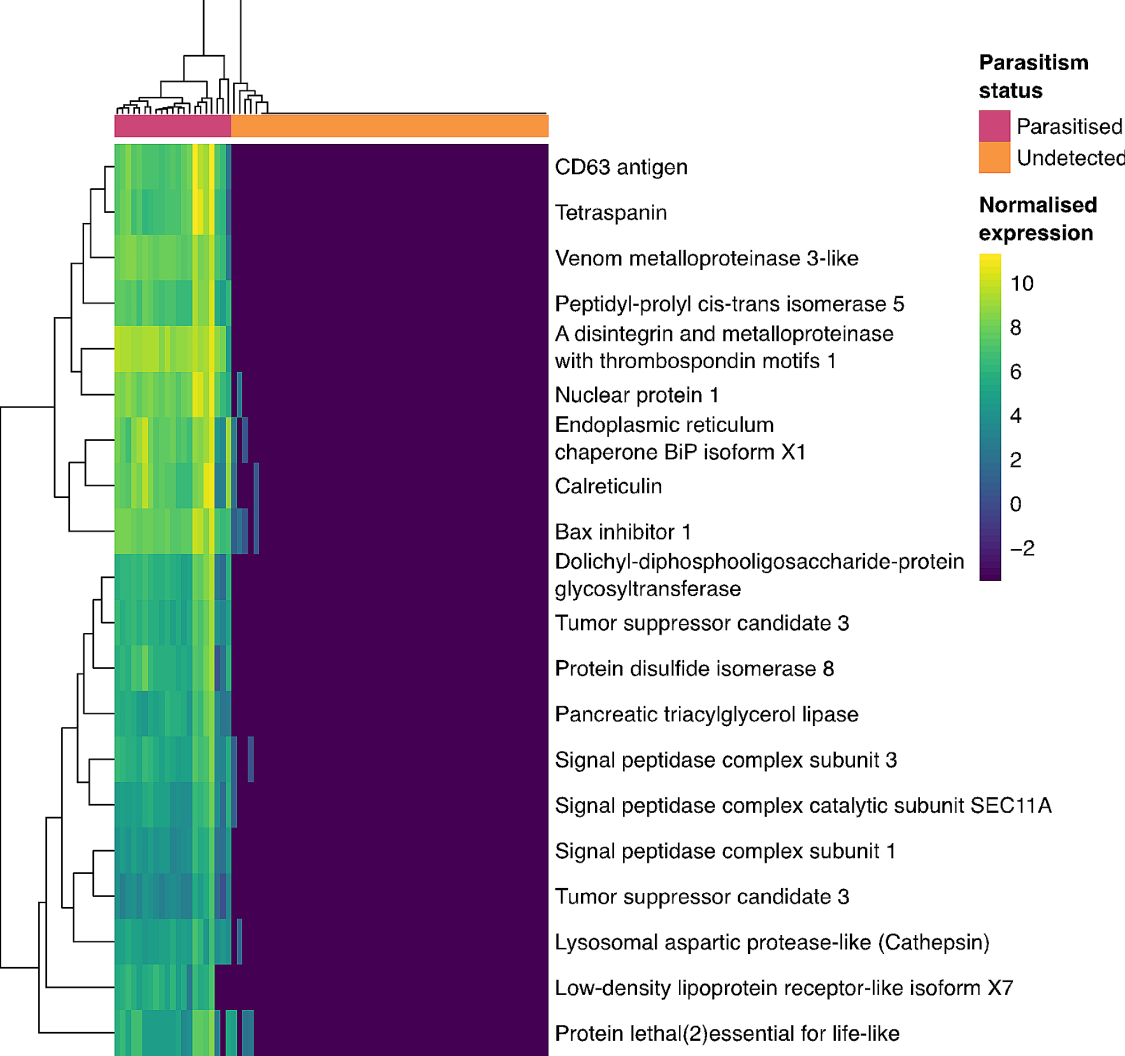
*M. hyperodae* venom components significantly expressed in parasitised ASW. A clustered heatmap showing VST normalized expression of venom DEGs with a mean TPM value above five and BlastX annotations. The parasitism status of each ASW sample is indicated in pink and orange boxes

We previously identified 64 candidate venom components in the *M. hyperodae* transcriptome, with annotation and TPM values above 200. Three of these were expressed in the ovaries and venom glands, while the rest had venom-specific expression patterns [15]. Continued expression of 36 of these candidate venom components was detected in parasitised ASW (Fig. 3, Supplementary Table 5). This continued venom expression inside parasitised ASW may be the result of venom gene expression by teratocytes (or their precursor cells before the egg hatching). *M. hyperodae* venom genes with continued expression in parasitised ASW include a lipase, cathepsin, calreticulin, metalloproteinases, and tetraspanins (Fig. 3).

The high expression of the *M. hyperodae* lipase, cathepsin and low-density lipoprotein receptor venom components in parasitised ASW likely links to the significant upregulation of ASW genes involved in lipid transport after parasitism. Lipases are a common venom component in other parasitoids, and cathepsin is involved in fat body degradation in *Spodoptera littoralis* following parasitism by *Bracon nigricans* [104]. Low-density lipoprotein receptors have also been detected in other parasitoid venoms [105–107] where they allow for the uptake of these mobilised lipids by the developing parasitoid. This suggests that these components may be involved in prolonged manipulation of the ASW host environment to provide lipids for the developing *M. hyperodae* egg.

In other host-parasitoid systems, calreticulin and metalloproteinases have roles in manipulating the host immune system to prevent recognition and response to the parasitoid egg; such as encapsulation or melanisation [105, 108–110]. Continued expression of these venom candidates in parasitised ASW may be preventing such a response, with calreticulin also expressed on the *M. hyperodae* egg surface [15].

There are two *M. hyperodae* venom candidates with high expression in parasitised ASW that belong to the tetraspanin family (tetraspanin and CD63 antigen). Tetraspanins are membrane proteins that, through binding to other tetraspanins or chaperone proteins, regulate processes such as intercellular immune interactions including cell adhesion and migration [111]. Tetraspanins can facilitate viral infection, through both viral entry to cells and in viral particle release [112], though are less well studied in the context of insect virus pathogenesis. In *Bombyx mori* expression of a particular tetraspanin is significantly increased following infection with the dsDNA virus *Bombyx mori* nucleopolyhedrovirus, with both overexpression and knockdown experiments showing that this expression promotes virus proliferation [113]. Given the transmission of MhFV to all parasitised ASW, high expression of tetraspanins in *M. hyperodae* venom glands [15] and by *M. hyperodae* in parasitised ASW, this tetraspanin expression may promote the proliferation of MhFV. No ASW tetraspanins were differentially expressed after parasitism.

### ASW differential gene and exon expression between locations with different historical parasitism rates & selection pressures

To determine the impact of ASW source location, and the associated variance in historical selective pressure strength on ASW gene expression, we carried out a pairwise comparison with DESeq2. This detected 84 significant DEGs, reduced to 23 after TPM filtering. Three of these DEGs had significant BlastX hits, all of which had higher expression in Ruakura ASW (Table 2). These annotated DEGs had relatively small log_2_ fold-changes (Table 2) which alongside the small number of filtered DEGs indicates that the difference in gene expression between ASW from Invermay and Ruakura is modest.

**Table 2:** ASW genes significantly differentially expressed in the location analysis and with BlastX annotation

Trinity Gene ID	LFC	Padj	BlastX Annotation	Sequence identity (%)	BlastX E-value	Invermay mean TPM	Ruakura mean TPM
TRINITY_DN114_c0_g1	2.68	5.17E-03	juvenile hormone esterase-like (*Leptinotarsa decemlineata*)	53.59	7.39E-117	124.88	21.96
TRINITY_DN19249_c0_g1	3.70	3.16E-04	pancreatic triacylglycerol lipase-like (*Sitophilus oryzae*)	46.02	7.64E-108	28.79	5.01
TRINITY_DN5987_c2_g1	2.75	5.36E-03	Retrovirus-related Pol polyprotein from transposon 412-like Protein (*Tribolium castaneum*)	49.05	0	18.84	3.44

DESeq2 Log_2_ fold-changes (LFC) and adjusted P-values (Padj) are reported from DESeq2 analysis, BlastX annotation is from a search against the non-redundant database, and mean TPM values for experimental groups were calculated from Salmon output. A positive LFC indicates higher expression in Invermay ASW

TRINITY_DN114_c0_g1 is annotated as a juvenile hormone esterase (Table 2), an enzyme required for the degradation of juvenile hormone, which regulates many physiological processes in insects including development, reproduction, diapause and metabolism [114–116]. TRINITY_DN19249_c0_g1 is annotated as a triacylglycerol lipase (Table 2) an essential enzyme for lipid metabolism. Triacylglycerol is one of the most important energy stores in insects required for many physiological processes in insects such as development, reproduction, and flight [117, 118]. TRINITY_DN5987_c2_g1 is annotated as a retrovirus-related Pol polyprotein from the long terminal repeat retrotransposon 412 (Table 2). This transposon is 7566 bp in length, compared to the 8704 bp assembled transcript, though the amino acid sequence identity is only 49%. Insertion sites for transposon 412 in *Drosophila simulans* are known to vary based on geographic location [119], with a negative correlation based on minimum temperature [120]. Higher expression in Ruakura ASW compared to Invermay fits this pattern.

ClusterProfiler detected significant enrichment of no Pfam domains and 24 GO terms in the DEG list, though for all significantly enriched GO terms, this enrichment was driven by only one or two DEGs. Investigation of these terms and the DEGs driving their enrichment did not reveal any putative parasitism resistance or avoidance mechanisms. These differential gene expression results suggest there is minimal difference in gene expression between ASW from Ruakura and Invermay when parasitism status is controlled for, with no clear link between detected differences and parasitism resistance or avoidance mechanisms.

DEXSeq analysis identified significant differential exon usage (DEU) based on ASW location in 7137 exons in 3094 genes, with only five of these genes also detected in the DESeq2 location analysis (Supplementary Table 6). Of those genes with significant DEU, 1253 had BlastX annotation from Trinotate. Annotations were involved in many processes, for example with 57 gene annotations derived from transposons (e.g., transposases, transposon polyproteins, RNA-directed DNA polymerases), 30 genes annotated as zinc finger proteins and 26 genes annotated as cytochrome P450s. However, overrepresentation analysis with ClusterProfiler only detected significant enrichment of four GO terms and three Pfam domains, with enrichment of these GO terms driven by a small percentage of the genes that had significant DEU (Supplementary Fig. 5). These enrichment results indicate that while there was a large amount of significant DEU between ASW locations, there was no significant enrichment towards certain processes, revealing no clear link to biocontrol decline.

### ASW differential gene and exon expression after parasitoid exposure

Given the potential for parasitoid exposure alone to elicit behavioural changes [121], which may or may not be related to parasitism resistance mechanisms, we investigated how such exposure impacts ASW gene expression. A pairwise comparison between all exposed ASW to all control samples (controlling for location-based differences) with DESeq2 detected 78 DEGs, which was reduced to only two after TPM filtering, one higher in exposed ASW and one in ASW not exposed to *M. hyperodae* (Supplementary Table 7). Neither had a significant BlastX hit from Trinotate or the non-redundant database. ClusterProfiler analysis was not performed on these DESeq2 results as there were only two genes with GO term or Pfam annotation in this DEG list.

DEXSeq analysis comparing ASW that were or were not exposed to *M. hyperodae* identified DEU in 16 exons contained in 15 genes, none of which were detected in the DESeq2 analysis. Seven of these exons were contained in six genes that had BlastX annotation from Trinotate (Supplementary Table 8). TRINITY_DN3053_c0_g1 was annotated as the metalloprotease Neprilysin-2, which in *Drosophila melanogaster* is involved in several processes during reproduction, affecting female fertility, egg laying, and embryogenesis [122], as well as being involved in middle and long-term memory formation [123]. However, this involvement in *D. melanogaster* memory formation is related to expression level rather than differential exon usage [123]. It is unclear what role the differential exon usage of Neprilysin-2 after parasitoid exposure has and whether this links to memory formation and/or behaviour. ClusterProfiler analysis on DEXSeq results detected significant enrichment of seven GO terms and four Pfam domains in the list of genes with significant DEU (Supplementary Fig. 6). The enrichment of each term/domain was driven by a single gene with significant DEU, with five genes total driving the enrichment of different terms/domains. None of the enriched terms or domains have clear links to insect memory or behaviour, nor any putative parasitism resistance/avoidance mechanisms.

An interaction analysis to investigate location-specific responses to parasitoid exposure with DESeq2 found 85 DEGs reduced to 20 by TPM filtering, three of which had significant BlastX hits (Table 3). Expression of TRINITY_DN29408_c0_g1, annotated as xanthine dehydrogenase, increased in Invermay ASW after exposure to *M.* hyperodae but decreased in Ruakura ASW after exposure (Table 3). Xanthine dehydrogenase has been shown to protect against reactive oxygen species and plays a role in the humoral immune response against bacteria in *Drosophila* [124, 125]. Expression of TRINITY_DN14828_c0_g1 was increased after exposure by Invermay ASW but decreased by Ruakura ASW and was annotated as blastoderm-specific protein 25D (Table 3) though has significant hits to Ninein in other insects (not shown). These genes are involved in microtubule stability, binding and anchoring at the centrosome, and play a role in *Drosophila* oogenesis [126]. TRINITY_DN3224_c0_g2 expression was increased after exposure to Ruakura ASW but decreased by Invermay ASW and was annotated as a reverse transcriptase domain-containing protein (Table 3). It is annotated as a reverse transcriptase (RNA-directed DNA polymerase). Insects do not encode their reverse transcriptases, relying on those from retrotransposons instead [127, 128]. None of the annotated DEGs from the location exposure interaction analysis have clear links to the alteration of insect behaviours. No genes in this DEG list had Pfam or GO term annotation, preventing ClusterProfiler analysis.

**Table 3:** ASW genes significantly differentially expressed in the *M. hyperodae* exposure-location interaction analysis, with BlastX annotation

Trinity Gene ID	Padj	BlastX Annotation	Sequence identity (%)	BlastX E-value	Exposed Dunedin mean TPM	Control Dunedin mean TPM	Exposed Ruakura mean TPM	Control Ruakura mean TPM
TRINITY_DN29408_c0_g1	3.12E-09	xanthine dehydrogenase-like isoform X2 (*Anthonomus grandis grandis*)	86.30	4.62E-37	14.76	0	2.18	5.17
TRINITY_DN14828_c0_g1	3.70E-03	blastoderm-specific protein 25D isoform X2 (*Sitophilus oryzae*)	79.12	1.48E-39	3.84	0	0	11.01
TRINITY_DN3224_c0_g2	6.81E-06	reverse transcriptase domain-containing protein, partial (*Listeria welshimeri*)	42.65	6.50E-15	0	3.95	6.72	0.15

The adjusted P-values (Padj) are reported from DESeq2 analysis, BlastX annotation is from a search against the non-redundant database, and mean TPM values for experimental groups were calculated from Salmon output

DEXSeq analysis investigating location-specific responses to parasitoid exposure had to be performed as pairwise comparisons between exposure treatment for each location separately, as DEU analysis requires that the final interaction term be condition:exon. The pairwise comparison with only Invermay ASW detected significant DEU for 229 exons in 168 genes, with 55 of these genes annotated by Trinotate BlastX (Supplementary Table 9). None of these genes were detected in the DESeq2 analysis, nor did their Blast or Pfam annotations have any clear links to insect behaviour.

The same analysis for Ruakura ASW detected significant DEU for 121 exons in 100 genes, with 39 of these genes annotated by Trinotate BlastX (Supplementary Table 10), none of which were detected in the DESeq2 analysis or the Invermay DEXSeq analysis. This list again contained the annotation Neprilysin-2 (TRINITY_DN692_c0_g1), though for a different Trinity gene than was detected in the DEXSeq exposed analysis with samples from both locations. ClusterProfiler did not detect significant overrepresentation of any GO terms or Pfam domains.

### ASW differential gene and exon expression based on *M. hyperodae* oviposition attempt status

Transcriptomic differences between ASW that did or did not have oviposition attempted by *M. hyperodae* were investigated to identify genes that may either play a role in determining susceptibility to parasitism and/or response to an oviposition attempt by *M. hyperodae*. Pairwise comparison with DESeq2 between ASW with observed oviposition attempts or not detected 13 DEGs, reduced to two after TPM filtering, both of which had significant BlastX hits (Table 4).TRINITY_DN2689_c1_g2, which had higher expression in ASW that had an observed oviposition attempt, was annotated as MNN4-like (Table 4), which in *S. cerevisiae* is involved in Mannosyl-phosphorylation of cell wall proteins [129]. There were better Blast hits for TRINITY_DN2689_c1_g2 from species within Insecta, though these were all to hypothetical or unnamed proteins providing no information about potential function. TRINITY_DN46396_c0_g1 had higher expression in ASW for which oviposition attempts were observed or inferred, and was annotated as translation elongation factor 2-like (Table 4), which has a role in protein synthesis [130]. As only a single gene had a Pfam domain annotation, and none had GO annotation this prevented overrepresentation analysis. DEXSeq analysis based on ASW oviposition attempt status detected significant DEU in two exons from two genes, one of which was annotated as a bacterial chaperonin. As there were only two genes with DEU, overrepresentation analysis was not performed.

**Table 4:** ASW genes significantly differentially expressed in the *M. hyperodae* oviposition attempt analysis and with BlastX annotation

Trinity Gene ID	LFC	Padj	BlastX Annotation	Sequence identity (%)	BlastX E-value	Oviposition attempted mean TPM	Not observed mean TPM
TRINITY_DN46396_c0_g1	22.55	8.80E-06	translation elongation factor 2-like (*Agrilus planipennis*)	91.67	9.57E-38	46.40	0
TRINITY_DN2689_c1_g2	-5.20	4.91E-02	protein MNN4-like (*Sitophilus oryzae*)	41.33	2.13E-09	5.45	23.96

DESeq2 Log_2_ fold-changes (LFC) and adjusted P-values (Padj) are reported from DESeq2 analysis, BlastX annotation is from a search against the non-redundant database, and mean TPM values for experimental groups were calculated from Salmon output. A positive LFC indicates higher expression in ASW that had oviposition attempted by *M. hyperodae*

An interaction analysis to identify location-specific gene expression differences between ASW oviposition attempt status found 15 DEGs, reduced to three by TPM filtering, with only TRINITY_DN15597_c3_g1 annotated, as a defensin (Table 5). While Ruakura ASW had higher expression of TRINITY_DN15597_c3_g1 when they had an oviposition attempt by *M. hyperodae*, Invermay ASW had higher expression when an oviposition attempt was not observed. However, this higher Invermay expression was primarily driven by a single sample with a TPM of 1008, which when removed reduces the average TPM for Invermay ASW from 96.5 to 13.6, only slightly higher than the TPM of 12.8 for Ruakura ASW with an oviposition attempt (Table 5). This may suggest that the outlier Invermay sample had an unsuccessful oviposition attempt that was not observed and couldn’t be inferred from parasitism status. Alternatively, the ASW may have been injured in another way causing increased expression of defensin. As none of the DEGs had Pfam or GO annotation overrepresentation analysis could not be performed.

**Table 5:** ASW genes significantly differentially expressed in the *M. hyperodae* oviposition attempt:location interaction analysis, with BlastX annotation

Trinity Gene ID	Padj	BlastX Annotation	Sequence identity (%)	BlastX E-value	Dunedin oviposition attempted TPM	Dunedin not observed TPM	Ruakura oviposition attempted mean TPM	Ruakura not observed TPM
TRINITY_DN15597_c3_g1	1.11E-02	Defensin (*Sitophilus zeamais*)	72.62	1.30E-34	12.77	96.49	37.43	6.13

The adjusted P-values (Padj) are reported from DESeq2 analysis, BlastX annotation is from a search against the non-redundant database, and mean TPM values for experimental groups were calculated from Salmon output

DEXSeq analysis investigating location-specific responses to parasitoid oviposition attempt status had to be performed as pairwise comparisons for each location separately. The comparison with only Invermay ASW detected significant DEU for 4 exons in 3 genes, two of which were annotated by Trinotate BlastX as a heat shock protein and a lipase (Supplementary Table 11), and none of which were detected in the DESeq2 interaction analysis. The comparison with only Ruakura ASW detected significant DEU for a single exon in one gene, which was not annotated by Trinotate BlastX or detected in the DESeq2 interaction analysis. Overrepresentation analysis was not performed on the location-specific results due to the small number of genes with significant DEU.

These results indicate there is minimal difference in gene expression and exon usage between ASW that did or did not have an oviposition attempt made on them by *M. hyperodae*, whether conserved between both locations or location-specific. None of the detected changes in expression were linked to a putative parasitism resistance or avoidance mechanism.

### Variant calling & GWAS

To identify genetic variation that might explain apparent resistance to parasitism, we performed variant calling and then a GWAS analysis. Variant calling on ASW supertranscripts and subsequent filtering resulted in 6,122 biallelic SNPs in 3,506 ASW genes, reduced to 4,275 SNPs by linkage pruning. Principal components analysis revealed that PC1 was explained by ASW location, accounting for 18.2% of the variation between samples (Supplementary Fig. 7). No further PCs were explained by experimental factors such as oviposition attempt status or parasitism. GWAS analyses did not detect any significant association with the oviposition attempt status or parasitism status of ASW, whether performed on the full unpruned, full pruned, Ruakura-only, or Invermay-only datasets. This inability to detect variants associated with oviposition attempt or parasitism status may be a result of small sample sizes resulting in inadequate power to detect such associations, particularly if parasitism avoidance/resistance involves many variants of small effect, as has been hypothesised previously [24].

## Discussion

### Multi-species RNA-seq reveals ASW responses to parasitism, as well as the transmission of MhFV and continued *M. hyperodae* venom expression within parasitised ASW

ASW parasitised by *M. hyperodae* are known to have some but not all internal organs consumed (with only the digestive system and some thoracic tissue remaining), have reduced flight capacity, and are reproductively sterilised soon after oviposition [6, 11, 12]. Significant changes in ASW expression of genes involved in glucose metabolism, lipid transport and muscle components likely relate to the need for the developing *M. hyperodae* egg to mobilise nutrients from the host for its nutrition. This potentially links to the reduced flight capability of parasitised ASW, particularly given the significant downregulation of flightin expression. Previous characterisation of *M. hyperodae* venom indicated the presence of several lipases, a venom acid phosphatase and angiotensin-converting enzyme-like which all have hypothesised roles in this nutritional sourcing [15], with continued expression of one *M. hyperodae* venom lipase detected in parasitised ASW. Manipulation of host lipids has been observed after parasitism in several species e.g. *Aphis gossypii* [131], likely again to reflect the parasitoid egg manipulating the host to provide nutrition while it develops.

Upregulated gene expression for innate immune system components by parasitised ASW, such as antimicrobial peptides, may act to prevent potential infection of the wound created by *M. hyperodae* oviposition, which would otherwise compromise the survival of the developing parasitoid egg. RNA-seq of *M. hyperodae* indicated their venom contains a GILT-like protein and a waprin-Thr1-like protein, both of which have putative roles in stimulating the innate immune system [15]. Expression of GILT-like is also continued in parasitised ASW (though with a TPM value below 5). Antibacterial activity of venom components has been previously detected in other parasitoid wasp species [132, 133].

No components of the cellular immune system involved in the encapsulation and melanisation response after parasitism were significantly upregulated in ASW after parasitism. *M. hyperodae* venom is known to contain various components that likely act to prevent this response, such as cathepsin and calreticulin, with the latter potentially deposited on the *M. hyperodae* egg surface [15], and both of which have continued expression in parasitised ASW. Though without standardised time points, and with most parasitised ASW from the Southern region where parasitism rates have not declined significantly, this is not a comprehensive investigation into this potential resistance mechanism.

Taking a multi-species approach to RNA-seq analysis revealed transmission of MhFV to all parasitised ASW. Whether or not MhFV infects ASW cells, or MhFV gene expression is confined to *M. hyperodae* cells within parasitised ASW has not been directly tested, and requires further investigation in future. Alongside MhFV transmission, is continued *M. hyperodae* venom expression inside parasitised ASW, with one of these venom components possibly facilitating virus replication. This viral transmission suggests that MhFV infection in *M. hyperodae* may play a role in the successful parasitism of ASW. *Leptopilina boulardi* eggs infected with a related virus, *Leptopilina boulardi* filamentous virus, are encapsulated significantly less by their *Drosophila* host [16], thus MhFV transmission during parasitism could be another factor modulating the ASW immune system during parasitism. MhFV infection has not been detected in any *M. aethiopoides* strains [35], and with *M. aethiopoides* parasitism of ASW also viable [134], MhFV infection is likely not necessary for successful parasitism of ASW by all *Microctonus* wasps.

MhFV expression was also detected in five unparasitised ASW samples in which PCR and subsequent analyses did not detect *M. hyperodae* expression, two of which had an observed and subsequently interrupted oviposition attempt. Previously, MhFV gene expression has also been detected in the RNA extracted from pooled heads of parasitised ASW, in which *M. hyperodae* tissue was not detected (data not included). This provides some indirect evidence that MhFV may also infect ASW cells, which should be tested directly in future, as the possibility of transmitting *M. hyperodae* cells expressing MhFV genes during unsuccessful oviposition attempts cannot be excluded. The transmission of MhFV to unparasitised ASW didn’t have a large effect on ASW gene expression when compared to unparasitised and uninfected ASW. However, with samples taken soon after observed oviposition attempts these are likely at a very early stage of putative MhFV infection. The effects of MhFV on either *M. hyperodae* or parasitised ASW are not currently known, therefore it is difficult to hypothesise potential effects on unparasitised ASW.

A premature mortality phenomenon of ASW exposed to *M. hyperodae*, but not parasitised by them, has been observed in the past. This mortality occurs too soon after parasitoid exposure to be attributable to successful parasitism by *M. hyperodae* [135–137]. It has been hypothesised that the mortality may be the result of a toxin-antitoxin system, where unsuccessful oviposition attempts by *M. hyperodae* may transmit a toxin, with this toxicity reversed by an ovarian extract during successful parasitism [137].

A toxin-antitoxin system also acts with the parasitoid *Asobara japonica* and its host, with interruption of oviposition behaviour after envenomation but before egg laying causing a high rate of mortality in their *Drosophila* host [138]. The toxic venom fraction was determined to be viral particles (though it was not determined whether these were an infectious virus or derived from an endogenous viral element) [139]. This raises the possibility that MhFV transmission during unsuccessful oviposition attempts could explain premature mortality of ASW exposed to *M. hyperodae*, requiring future experiments to isolate the virus and inject it into ASW, and to verify whether MhFV infects ASW tissues. If MhFV does cause this premature mortality phenomenon in ASW, future work to investigate the prevalence of MhFV in *M. hyperodae* and to introduce it to uninfected wasps/increase MhFV load in infected wasps may assist in increasing biocontrol efficacy.

### Differential expression analyses did not identify a clear mechanism behind avoidance behaviours or biocontrol decline

Differential gene expression analysis revealed that there was only a modest difference in gene expression between Northern and Southern ASW populations when controlling for oviposition attempt and parasitism status. Differential exon usage analysis indicated larger differences in exon expression between both locations. No differences detected between these two populations, which have experienced different intensities of selective pressure imposed by *M. hyperodae*, indicated any parasitism resistance or avoidance mechanism. While these locations were used to represent resistant and susceptible weevils, this is only true at the population level, with parasitism rates at Ruakura indicating some weevils are still susceptible to parasitism. These two populations also represent extremes of the genetic cline of ASW in Aotearoa New Zealand [24], so these differences in gene and/or exon expression do not necessarily relate to biocontrol decline. Performing PCA on biallelic SNPs from RNA-seq data variant calling revealed that 18.2% of the variation between samples was explained by ASW location, consistent with this genetic cline.

There is a negligible gene expression response by ASW to *M. hyperodae* exposure which is conserved between both locations. Location-specific responses to *M. hyperodae* exposure are larger, both for gene expression and exon usage. This may reflect the variance in associated historical selective pressure to evolve resistance or avoidance mechanisms between the two locations. Avoidance behaviours in ASW, which are significantly more frequent in Northern and Central ASW populations, are also significantly more frequent when *M. hyperodae* is present [33]. There is no clear link between this behavioural alteration and the location-specific gene and exon expression responses. While unrelated to parasitism avoidance, transcriptomic analysis of females from five *Drosophila* species, which alter their mating behaviour when exposed to several parasitoid species, revealed changes in genes related to the immune system, stress response, and a micropeptide gene, with deletion of the latter preventing the behavioural alteration [121], indicating that parasitoid exposure can alter host behaviour through influencing gene expression and that these expression changes can be detected with RNA-seq.

Consistent with a previous genomic investigation of ASW biocontrol decline, we segregated weevil samples based on their location (and associated historical parasitism rates and selective pressure) and detected parasitism status [24]. To strengthen the phenotyping of ASW as resistant or susceptible to parasitism they were also observed in microcosms with *M. hyperodae*, and any avoidance behaviours or oviposition attempts were recorded. Despite this improved phenotyping, this analysis found a negligible difference between ASW where oviposition was attempted (considered to be susceptible) and ASW exhibiting avoidance behaviours where no oviposition attempts were observed or inferred from parasitism status (considered to be possibly resistant). This is despite analyses being performed to detect both gene expression changes that are conserved between both locations as well as location-specific responses. No changes in gene expression linked to putative resistance or avoidance mechanisms, despite our experiment being adequately powered to detect significant differences in gene expression if they existed. GWAS analyses also did not detect any variants associated with any experimental factors.

Many reasons could explain why we did not detect differential expression indicating a clear mechanism behind biocontrol decline. These include the following: *i*. the mechanism may not be associated with changes in gene or exon expression in ASW *ii.* we may have detected expression differences associated with resistance/avoidance mechanisms in genes that lacked annotation, precluding identification of potential mechanisms, *iii*. we may have not detected changes in gene expression of lowly expressed genes involved in resistance/avoidance mechanisms, *iv*. there may be different mechanisms behind the different avoidance behaviours that have been characterised in ASW, *v.* regions associated with resistance/avoidance mechanisms may not have been assembled in the transcriptome (e.g., poly(A) enrichment may have depleted reads from endosymbionts that protect against parasitism).

## Conclusions

These analyses revealed the transcriptomic response of ASW to parasitism by *M. hyperodae*, involving modulation of the innate immune system, muscle components and lipid and glucose metabolism. Alongside this, we show continued expression of some *M. hyperodae* venom components inside parasitised ASW, as well as transmission of MhFV to parasitised ASW. We also detected MhFV in five ASW where parasitism could not be detected, including two where oviposition attempts were observed and interrupted. This MhFV transmission to unparasitised ASW may link to a premature mortality phenomenon in ASW exposed to but not parasitised by *M. hyperodae*. Building on a previous genomic analysis of ASW populations with genotyping-by-sequencing, these transcriptomic analyses also aimed to investigate potential mechanisms to explain the decline of ASW biocontrol by *M. hyperodae*. Despite strengthened phenotyping of ASW, based not only on their parasitism status but also their observed behaviour and oviposition attempt status in microcosms, we were unable to identify any potential resistance/avoidance mechanism/s.

### Electronic supplementary material

Below is the link to the electronic supplementary material.

Supplementary Material 1

Supplementary Material 2

## Data Availability

Raw sequence data from all samples used in the analysis are hosted at the National Center for Biotechnology Information (NCBI) Sequence Read Archive database with the accession PRJNA841752 for the exposure dataset, and PRJNA841759 for the microcosm dataset. The length-filtered ASW transcriptome assembly is hosted at the NCBI Transcriptome Shotgun Assembly (TSA) database under the PRJNA841752 accession. This assembly has a reduced number of transcripts after those labelled as contaminated during the submission process were removed. The M. hyperodae transcriptome and MhFV genome are available from prior publications with respective accessions of PRJNA841753 and OQ439926 [15,35]. Scripts used for analyses are hosted in GitHub repositories as specified in the methods.
