# Supplementary material for: Immune system modulation & virus transmission during parasitism identified by multi-species transcriptomics of a declining insect biocontrol system
Source: BMC Genomics. 2024 Mar 26;25:311. doi: 10.1186/s12864-024-10215-3 (PMC10964624; doi:10.1186/s12864-024-10215-3)
Supplement: Supplementary file 1 — Supplementary Material 1 [file 12864_2024_10215_MOESM1_ESM.pdf]

**Supplementary information**

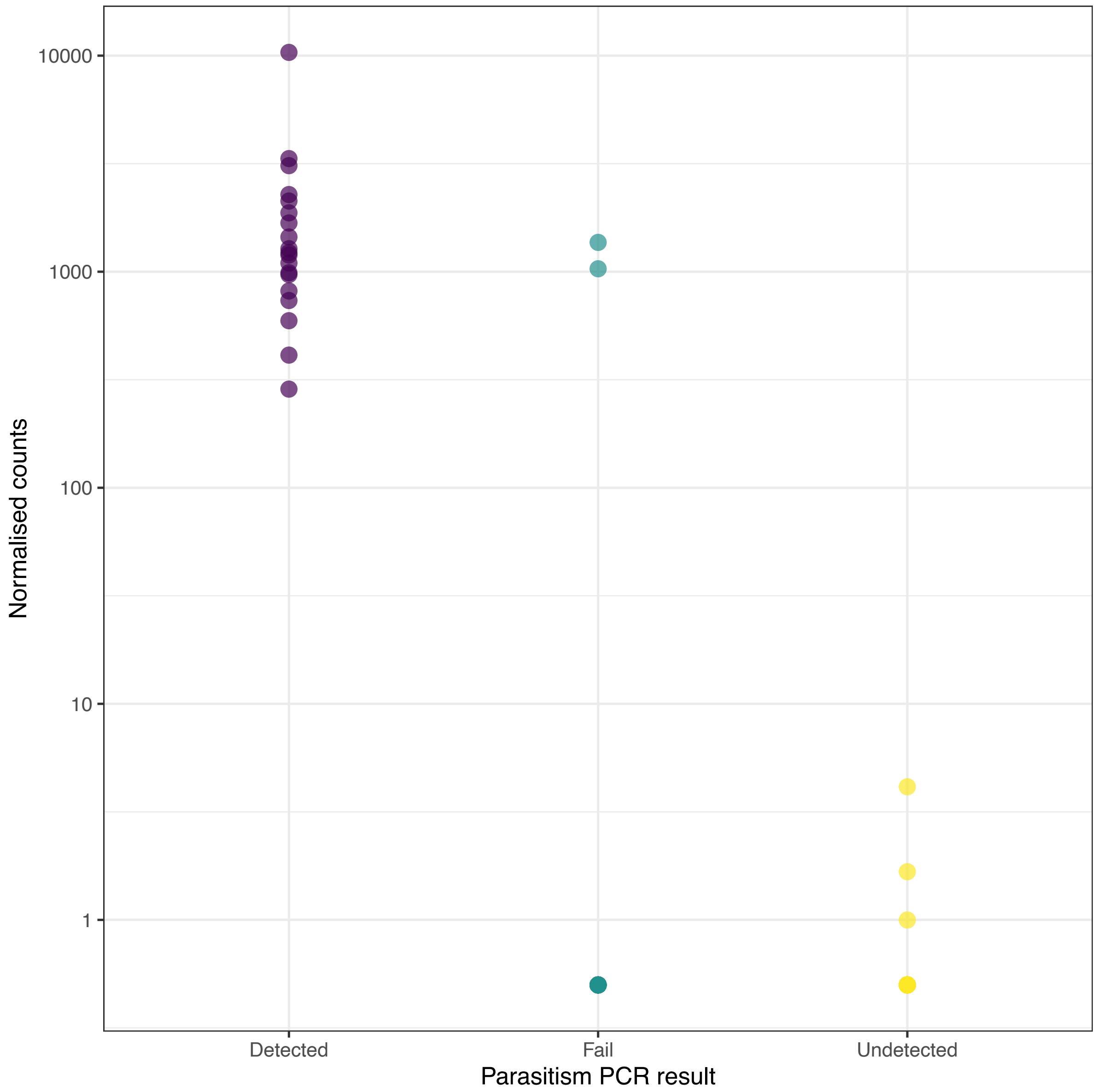


**Supplementary Figure 1** - A scatterplot displaying DESeq2 normalized counts against the *M. hyperodae* parasitism PCR target gene, TRINITY_DN481_c1_g1, on a Log10 scale. Samples are grouped based on their result in the multiplex parasitism RT-PCR result.

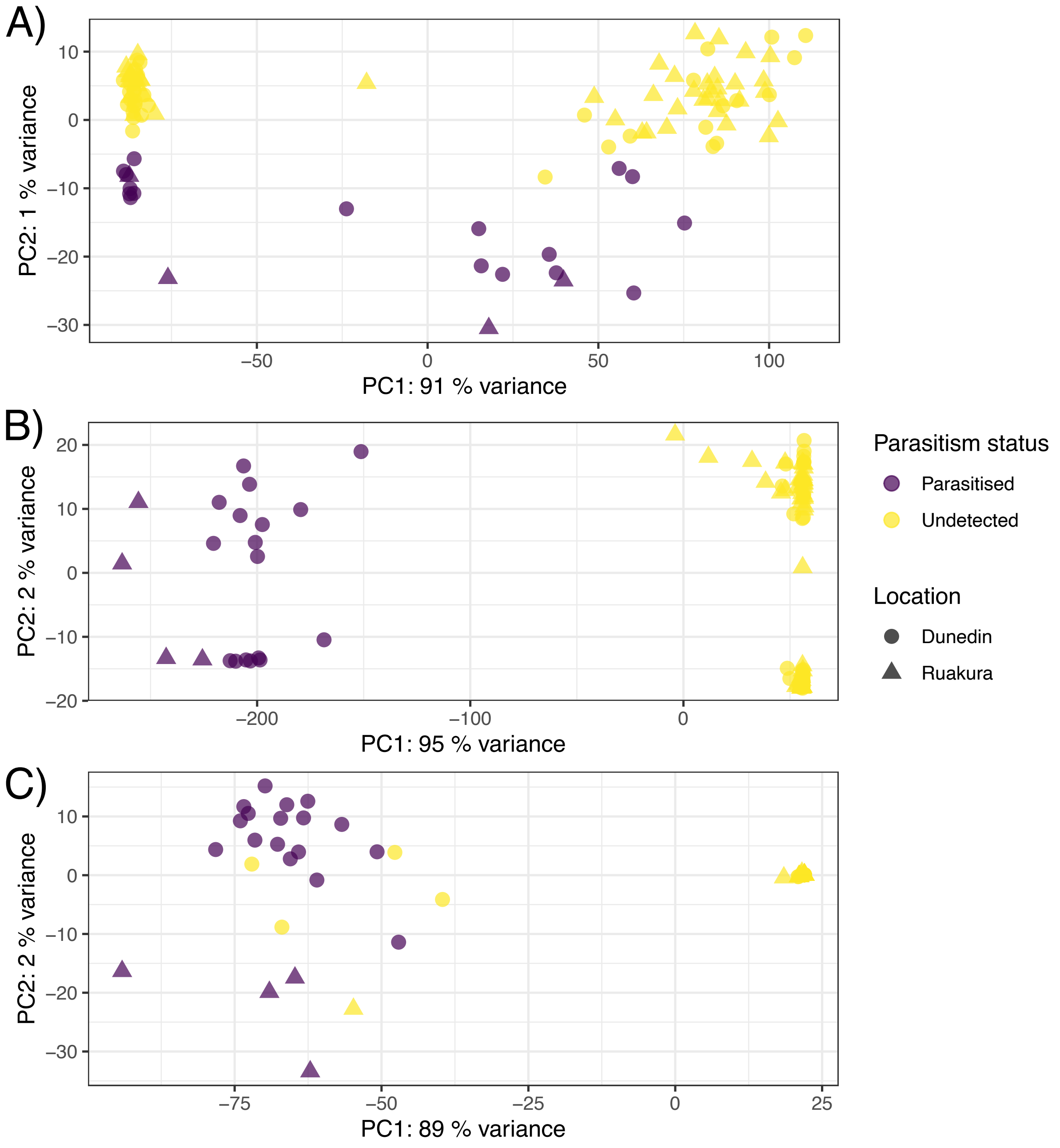


**Supplementary Figure 2** - Principal component analyses for all RNA-seq samples based on A) ASW, B) *M. hyperodae* and C) MhFV gene expression. Points are coloured based on their parasitism status and shaped based on ASW source location.

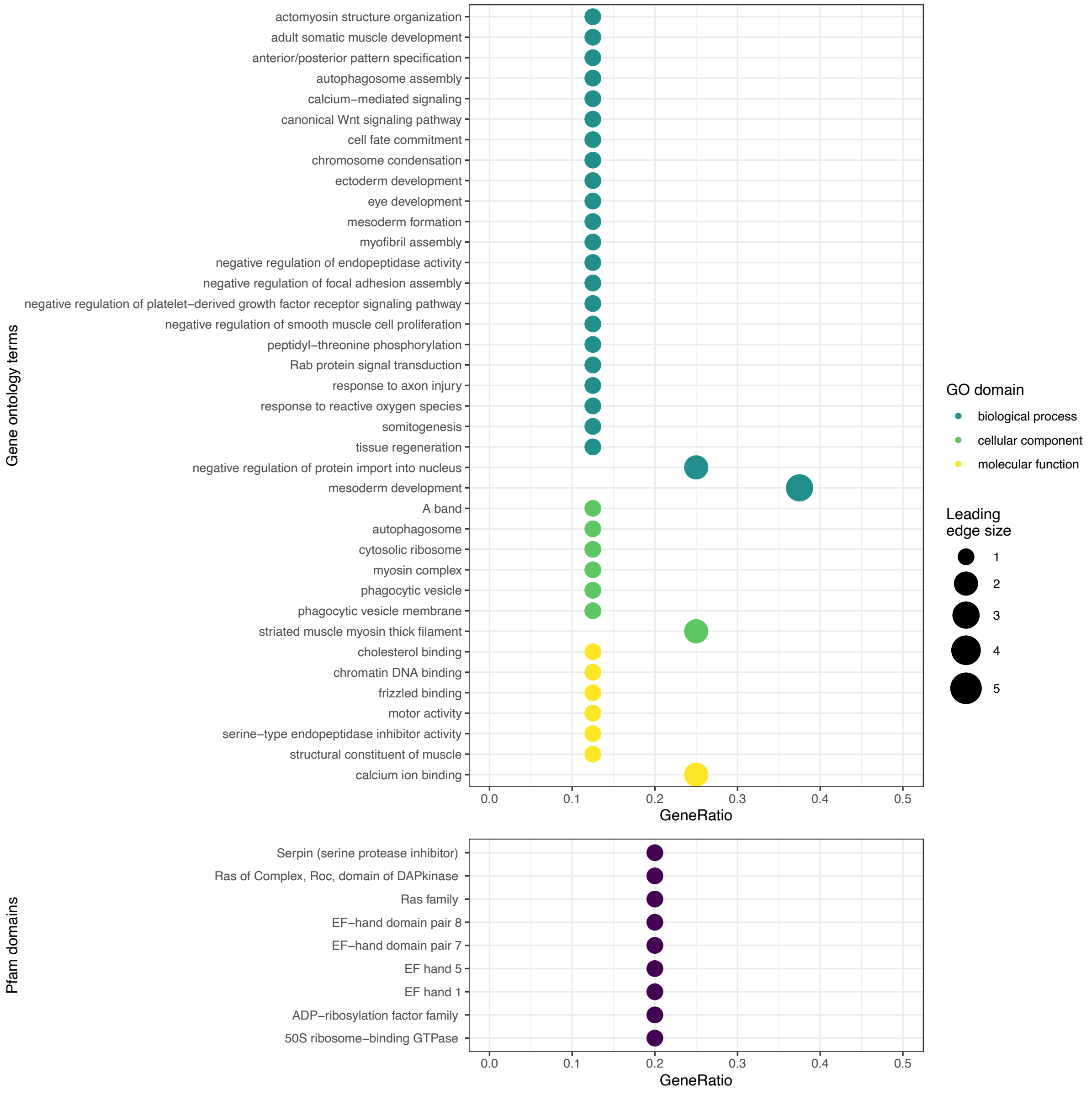


**Supplementary Figure 3** – Gene ontology and Pfam enrichment results from ClusterProfiler for ASW parasitism by *M. hyperodae* differential gene expression results. The GeneRatio is the ratio of the total differentially expressed genes with a GO /Pfam annotation to those with the associated GO term/Pfam domain. Points are sized based on the number of genes contributing to the enrichment of each term (leading edge).

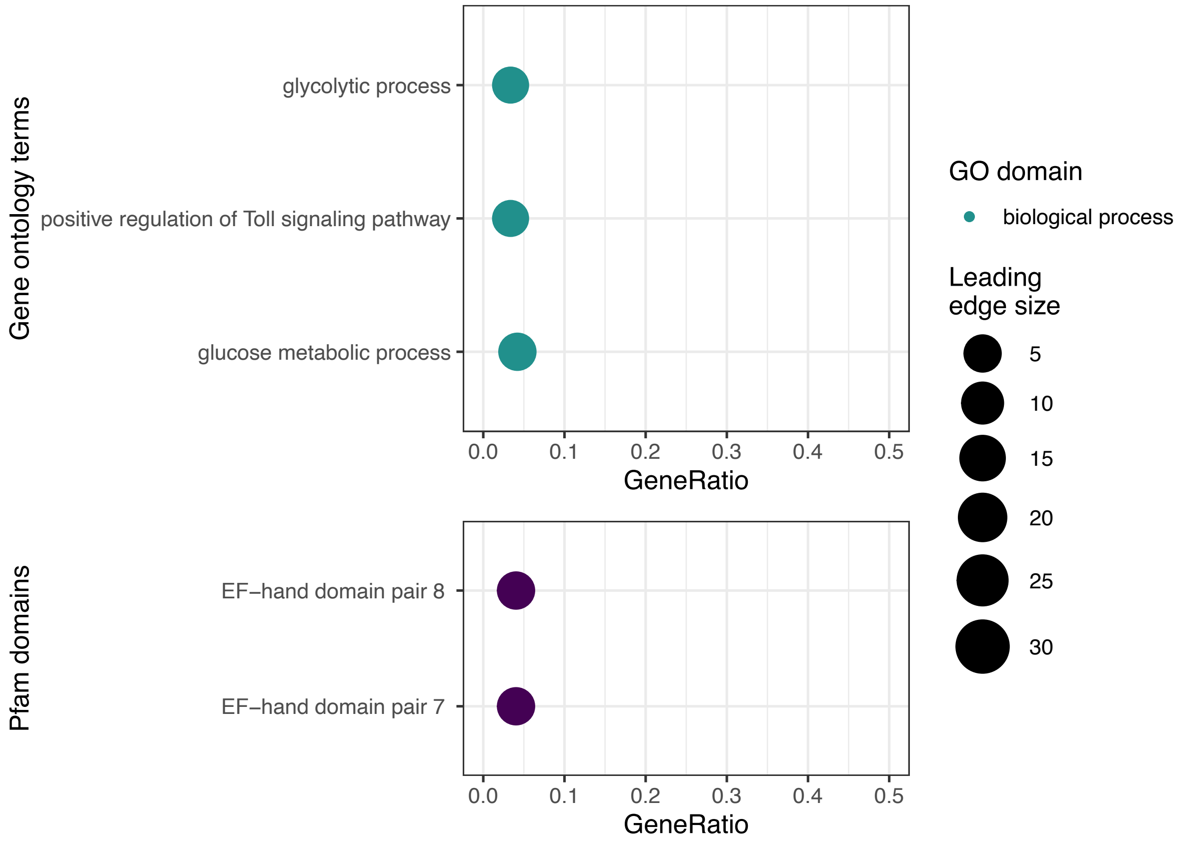


**Supplementary Figure 4** – Gene ontology and Pfam enrichment results from ClusterProfiler for ASW parasitism by *M. hyperodae* differential exon usage results. Points are sized based on the number of genes contributing to the enrichment of each term (leading edge), and sized based on their adjusted P-values.

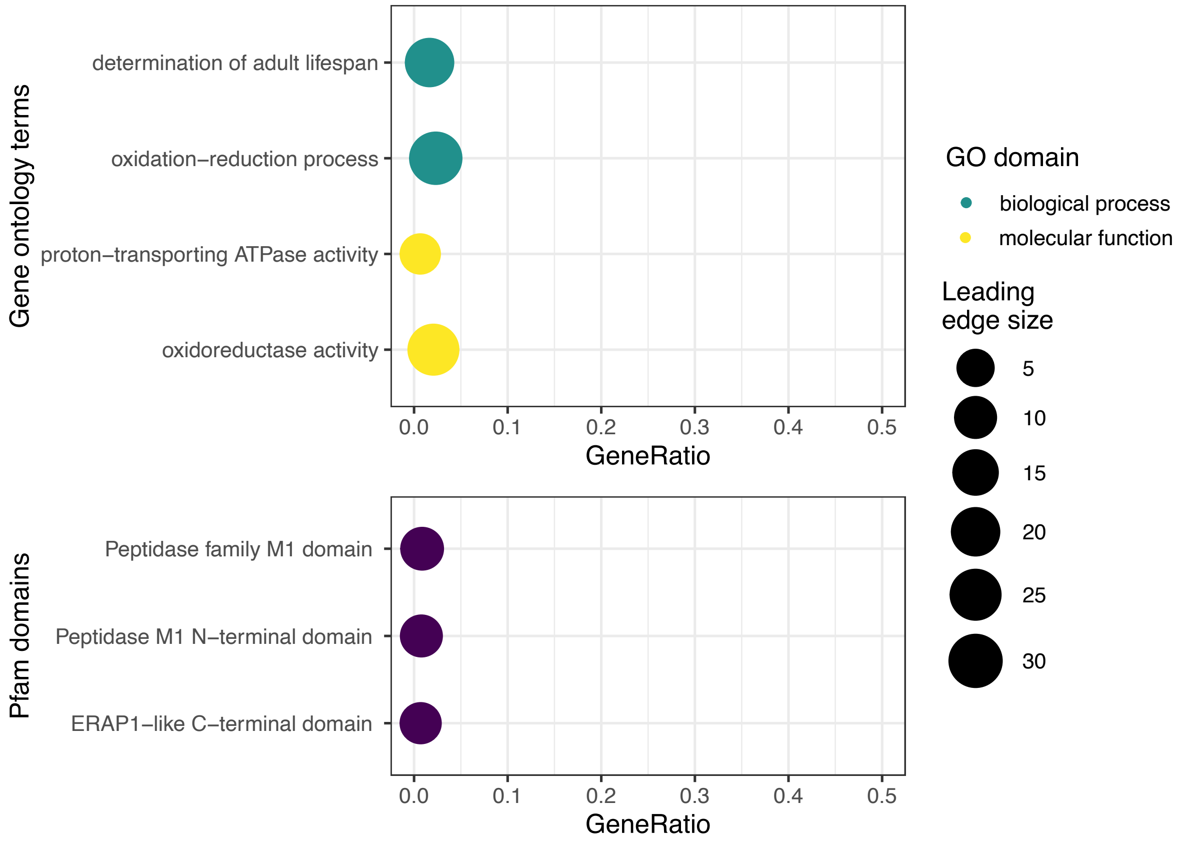


**Supplementary Figure 5** – Gene ontology and Pfam enrichment results from ClusterProfiler for ASW location differential exon usage results. Points are sized based on the number of genes contributing to the enrichment of each term (leading edge), and sized based on their adjusted P-values.

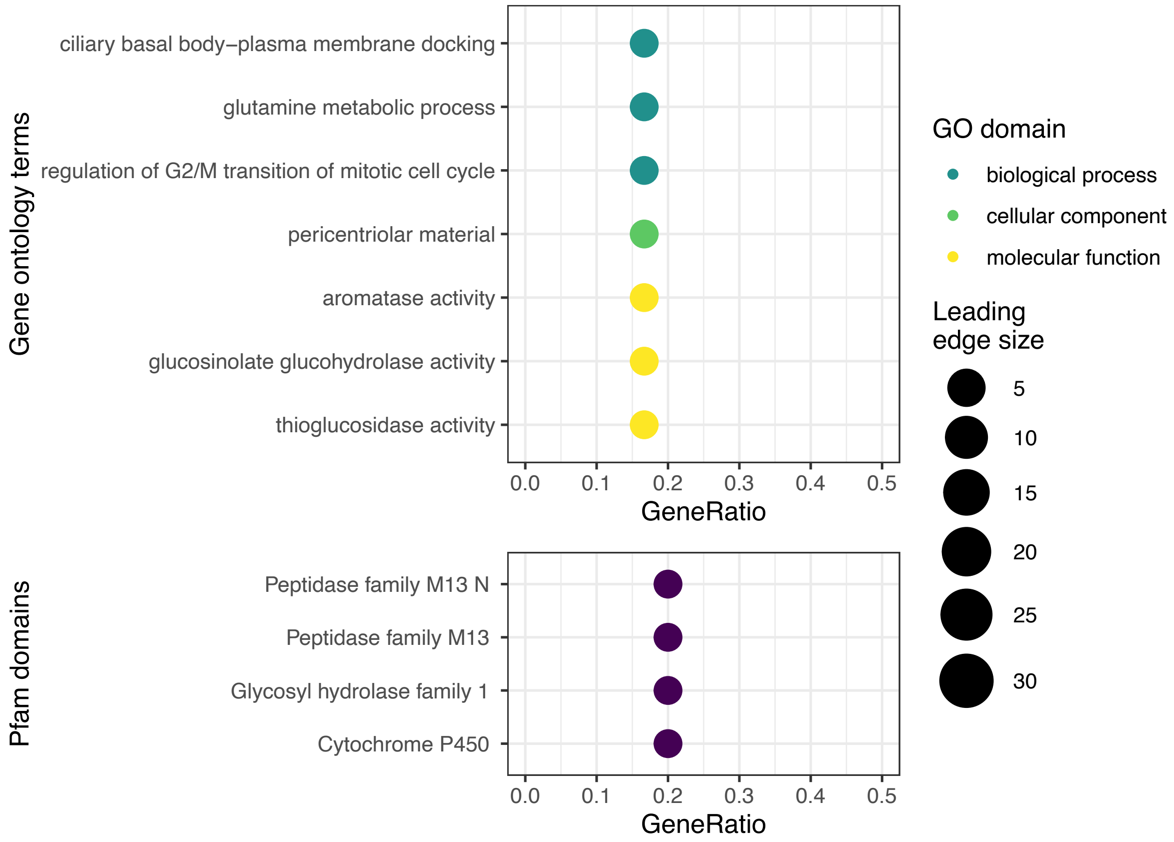


**Supplementary Figure 6** – Gene ontology and Pfam enrichment results from ClusterProfiler for ASW exposure to *M. hyperodae* differential exon usage results. Points are sized based on the number of genes contributing to the enrichment of each term (leading edge), and sized based on their adjusted P-values.

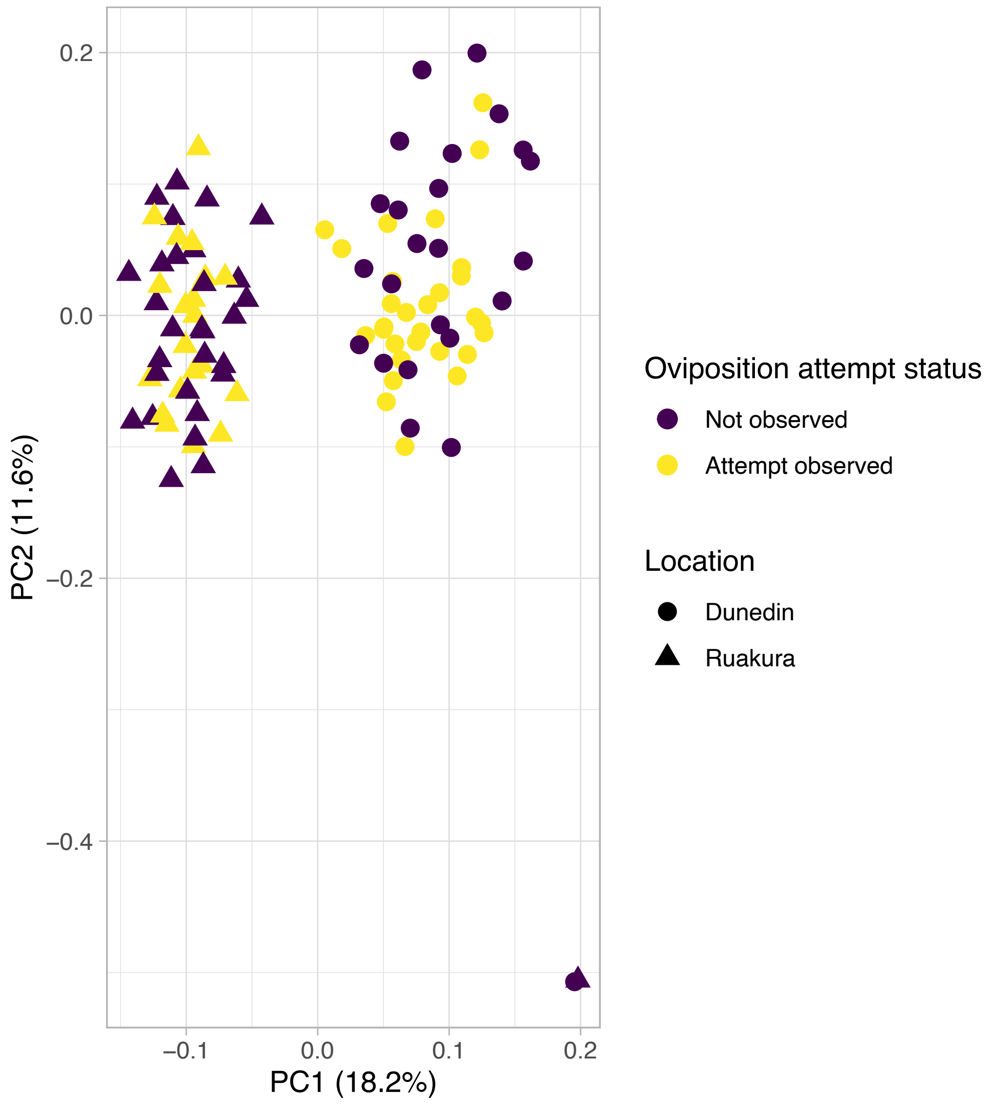


**Supplementary Figure 7** - Principal component analysis for all RNA-seq samples based on biallelic SNPs in ASW supertranscripts, after linkage pruning. Points are coloured based on *M. hyperodae* attack status, and shaped based on ASW source location.

**Supplementary Table 1** – ASW Microcosm RNA-seq sample groups when refined based on parasitism status.

| ASW Location | Group | Number of samples |
|---|---|---|
| Invermay | Successfully parasitised | 17 |
| | Oviposition observed & interrupted | 12 |
| | Oviposition not observed | 12 |
| Ruakura | Successfully parasitised | 4 |

| | | |
|---|---|---|
| | Oviposition observed & interrupted | 16 |
| | Oviposition not observed | 18 |

**Supplementary Table 2** – Power analysis results for ASW differential gene expression analyses.

| Comparison | Power when LFC threshold is 2 (%) | Power when LFC threshold is 5 (%) | Power when LFC threshold is 10 (%) | Samples from experiment/s |
|---|---|---|---|---|
| Location | 92.52 | 99.98 | 100.00 | Exposure & Microcosm |
| Exposure | 40.20 | 91.14 | 97.59 | Exposure |
| Ruakura exposed vs others | 39.00 | 90.40 | 97.18 | Exposure |
| Invermay exposed vs others | 37.84 | 89.99 | 97.15 | Exposure |
| Oviposition attempt status | 90.09 | 99.87 | 100.00 | Microcosm |
| Ruakura oviposition attempted vs others | 89.14 | 99.66 | 100.00 | Microcosm |
| Invermay oviposition attempted vs others | 89.55 | 99.78 | 100.00 | Microcosm |
| Parasitism | 90.13 | 99.87 | 100.00 | Microcosm |

**Supplementary Table 3** – Significant results from DEXSeq analysis of ASW parasitism. Trinotate Blast annotation results are presented in a single column, where Q: indicates query alignment range, H: indicates subject alignment range, %ID indicates sequence identity (%), and E: indicates E-value.

For Supplemental tables 4-11 see supplemental data file.

**Supplementary Table 4** – Significant results from DESeq2 analysis of MhFV expression in parasitised ASW. BlastP and Pfam annotations are reported from Inwood, Skelly, et al., (2023).

**Supplementary Table 5**– Significant results from DESeq2 analysis of *M. hyperodae* venom expression in parasitised ASW. BlastX and Pfam annotations, and venom TPM and ranks are reported from Inwood, Harrop et al., (2023).

**Supplementary Table 6** – Significant results from DEXSeq analysis of ASW source location. Trinotate Blast annotation results are presented in a single column, where Q: indicates query alignment range, H: indicates subject alignment range, %ID indicates sequence identity (%), and E: indicates E-value.

**Supplementary Table 7** – Significant results from DESeq2 analysis of ASW exposure to *M. hyperodae*. BlastX annotation is presented from a search against the non-redundant database, independent of Trinotate.

**Supplementary Table 8** – Significant results from DEXSeq analysis of ASW exposure to *M. hyperodae*. Trinotate Blast annotation results are presented in a single column, where Q: indicates query alignment range, H: indicates subject alignment range, %ID indicates sequence identity (%), and E: indicates E-value.

**Supplementary Table 9** – Significant results from DEXSeq results from Invermay ASW exposure to *M. hyperodae.* Trinotate Blast annotation results are presented in a single column, where Q: indicates query alignment range, H: indicates subject alignment range, %ID indicates sequence identity (%), and E: indicates E-value.

**Supplementary Table 10** – Significant results from DEXSeq analysis of Ruakura ASW exposure to *M. hyperodae*. Trinotate Blast annotation results are presented in a single column, where Q: indicates query alignment range, H: indicates subject alignment range, %ID indicates sequence identity (%), and E: indicates E-value.

**Supplementary Table 11** – Significant results from DEXSeq analysis of Invermay ASW oviposition status. Trinotate Blast annotation results are presented in a single column, where Q: indicates query alignment range, H: indicates subject alignment range, %ID indicates sequence identity (%), and E: indicates E-value.
